# Nutritional regulation of oligodendrocyte differentiation regulates perineuronal net remodeling in the median eminence

**DOI:** 10.1016/j.celrep.2021.109362

**Published:** 2021-07-13

**Authors:** Sara Kohnke, Sophie Buller, Danae Nuzzaci, Katherine Ridley, Brian Lam, Helena Pivonkova, Marie A. Bentsen, Kimberly M. Alonge, Chao Zhao, John Tadross, Staffan Holmqvist, Takahiro Shimizo, Hannah Hathaway, Huiliang Li, Wendy Macklin, Michael W. Schwartz, William D. Richardson, Giles S.H. Yeo, Robin J.M. Franklin, Ragnhildur T. Karadottir, David H. Rowitch, Clemence Blouet

**Affiliations:** 1 MRC Metabolic Diseases Unit, University of Cambridge Metabolic Research Laboratories, WT-MRC Institute of Metabolic Science, University of Cambridge, Cambridge CB2 0QQ, UK; 2 Department of Paediatrics and Wellcome-MRC Cambridge Stem Cell Institute, University of Cambridge, Cambridge, UK; 3 Wellcome-MRC Cambridge Stem Cell Institute, Department of Clinical Neurosciences, University of Cambridge, Cambridge, UK; 4 Wellcome-MRC Cambridge Stem Cell Institute, Department of Veterinary Medicine, University of Cambridge, Cambridge, UK; 5 Department of Cell & Developmental Biology and Program in Neuroscience, University of Colorado School of Medicine, Aurora, CO, USA; 6 Wolfson Institute for Biomedical Research, University College London, London, UK; 7 University of Washington Medicine Diabetes Institute, Department of Medicine, University of Washington, Seattle, WA, USA

**Keywords:** hypothalamus, energy balance, oligodendrocyte, median eminence, plasticity, nutrition, obesity, glucose homeostasis, perineuronal nets

## Abstract

The mediobasal hypothalamus (MBH; arcuate nucleus of the hypothalamus [ARH] and median eminence [ME]) is a key nutrient sensing site for the production of the complex homeostatic feedback responses required for the maintenance of energy balance. Here, we show that refeeding after an overnight fast rapidly triggers proliferation and differentiation of oligodendrocyte progenitors, leading to the production of new oligodendrocytes in the ME specifically. During this nutritional paradigm, ME perineuronal nets (PNNs), emerging regulators of ARH metabolic functions, are rapidly remodeled, and this process requires myelin regulatory factor (*Myrf*) in oligodendrocyte progenitors. In genetically obese *ob/ob* mice, nutritional regulations of ME oligodendrocyte differentiation and PNN remodeling are blunted, and enzymatic digestion of local PNN increases food intake and weight gain. We conclude that MBH PNNs are required for the maintenance of energy balance in lean mice and are remodeled in the adult ME by the nutritional control of oligodendrocyte differentiation.

## Highlights

• The median eminence contains a dense population of newly formed oligodendrocytes
• Nutritional signals regulate oligodendrocyte differentiation in the median eminence
• Oligodendrocyte plasticity in the median eminence regulates local perineuronal nets

## Graphical abstract

**Figure:**
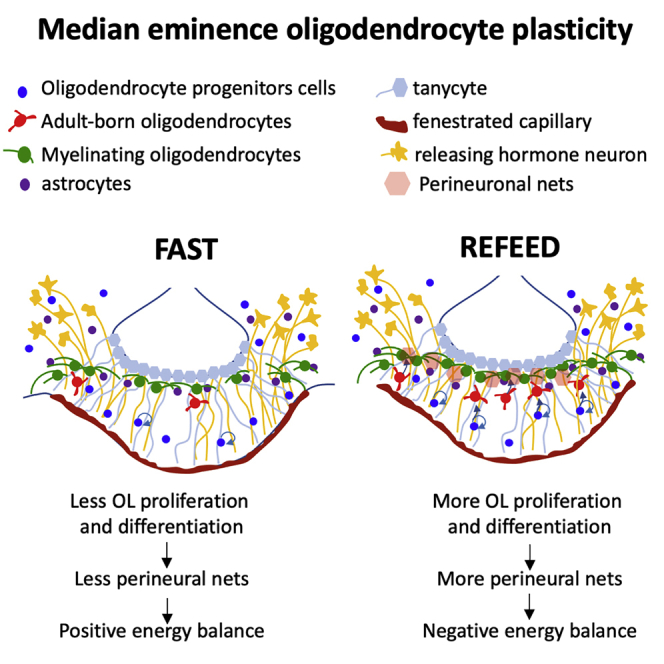

## Teaser

Kohnke et al. find that median eminence oligodendrocyte proliferation and differentiation uniquely respond to nutritional signals, revealing the plasticity of this cell type. They directly implicate adult-born oligodendrocytes in the regulation of local perineuronal nets,and show that these extracellular structures modulate the control of energy balance.

## Introduction

The median eminence (ME) of the mediobasal hypothalamus (MBH) is a bidirectional gateway between the hypothalamus and the periphery, with diverse roles in mammalian physiology and the regulation of neuroendocrine axes. The ME vasculature lacks a blood-brain barrier (BBB), allowing axons of hypothalamic neuroendocrine neurons to access a BBB-free area when entering the ME and release hypothalamic-releasing hormones into the portal circulation. The ME fenestrated endothelium also allows circulating signals to freely diffuse into the ME and adjacent arcuate nucleus of the hypothalamus (ARH), which is rich in neurons critical to appetite regulation and energy balance, thus giving local neurons privileged access to peripheral signals.

Tanycytes of the ME have been proposed to control the diffusion and transport of blood-borne signals from the ME to the adjacent ventricular space (ME-cerebrospinal fluid [CSF] barrier) (Langlet et al., 2013b). The ME-ARH barrier is functionally equally critical, but its structural components remain unclear (Yoo et al., 2019). Little is known about how other glial cell types may contribute to these functions, but emerging evidence highlights the unique properties of ME glial populations, in particular oligodendrocyte progenitor cells (OPCs). OPCs of the ME proliferate at a very high rate (Robins et al., 2013; Zilkha-Falb et al., 2020) and may continuously differentiate to oligodendrocytes (Chen et al., 2017; Pastor et al., 1991). Additional local roles for OPCs may also include the modulation of hypothalamic leptin sensing (Djogo et al., 2016; Yasumoto et al., 2018).

Emerging evidence indicates that the ME is highly plastic and rapidly responds to hormonal and nutritional signals (Clasadonte and Prevot, 2018; Parkash et al., 2015). This could relate to its unique unbuffered access to blood-borne signals. Hormonal and nutritional sensing in the ME initiate rapid structural remodeling, leading to changes in the ARH territory sitting outside the BBB and exposed to peripheral signals (Langlet et al., 2013a). ME tanycytes have been proposed to mediate the nutritional regulation of the ME-CSF barrier (Langlet et al., 2013a), but the mechanisms mediating the plasticity of the ME-ARH barrier in response to nutritional cues remain unclear.

Here, we report a role for oligodendrocyte lineage cells of the ME in response to nutritional signals. We found that the adult ME is rich in newly formed premyelinating oligodendrocytes expressing the specific marker *Bmp4*. Nutritional signals rapidly activate oligodendrocyte differentiation specifically in the ME and regulate the formation of perineuronal nets (PNNs), with rapid remodeling of these extracellular structures during the transition from the fasted to the refed state. Collectively, these studies reveal the unsuspected plasticity of ME oligodendrocytes in response to nutritional signals and its consequence on the remodeling of PNNs, an emerging key regulator of ARH metabolic sensing (Alonge et al., 2020; Mirzadeh et al., 2019).

## Results

### Single-cell transcriptomic (scRNA-seq) analysis reveals three types of oligodendrocyte lineage cells in the ME

**Figure 1:**
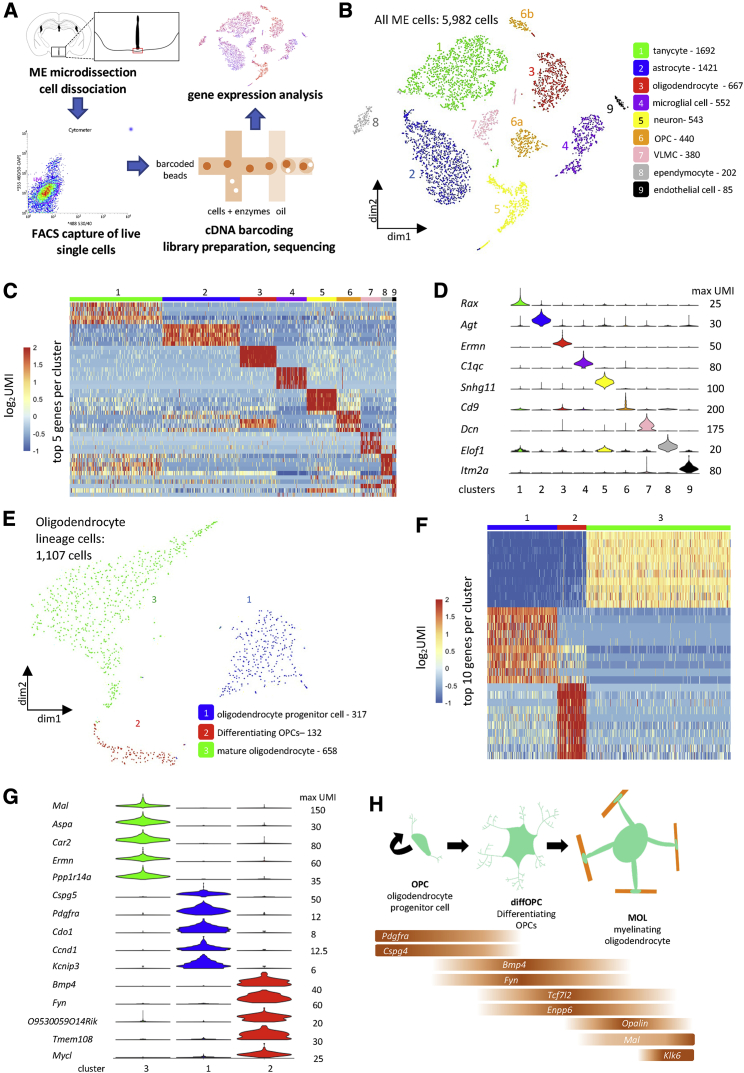
Single-cell transcriptomic analysis reveals 3 types of oligodendrocyte lineage cells in the ME (A) Workflow for single-cell RNA sequencing experiment. (B) tSNE plot demonstrating 9 groups of ME cells. OPCs, oligodendrocyte progenitor cells; VLMCs, vascular and leptomeningeal cells. (C) Heatmap of log_2_UMI (unique molecular identifier) counts per cell for the top 5 differentially expressed genes per cluster. (D) Violin plot showing UMI count distribution of one defining gene per cluster (variable scales per gene). (E) tSNE plot demonstrating 3 groups of oligodendrocyte lineage cells. (F) Heatmap of log_2_UMI counts per cell for the top 10 differentially expressed genes per cluster. (G) Violin plot showing UMI count distribution of the top 5 genes per cluster (variable scales per gene). (H) Schematic of oligodendrocyte differentiation and maturation and expression of validated substage markers in our oligodendrocyte lineage cells.

We used scRNA-seq to build a high-resolution cellular and molecular characterization of the adult mouse ME and to understand the transcriptional changes occurring during a nutritional transition from the fasted to the fed state (Figures 1A and S1A–S1C). We mapped each of the 5,982 sequenced cells onto a t-distributed stochastic neighbor embedding (tSNE) plot based on gene expression and identified 9 distinct cell clusters by using K-means clustering (Figure 1B; Figure S1D). We obtained the unique transcriptional signature of each cluster (Figure 1C; Table S1) and a defining gene for each cluster, as follows: tanycytes (*Rax*^+^, cluster 1), astrocytes (*Agt*^+^, cluster 2), oligodendrocytes (*Ermn*^+^, cluster 3), microglial cells (*C1qc*^+^, cluster 4), neurons (*Snhg11*^+^, cluster 5), OPCs (*Cd9*^+^, cluster 6), vascular and leptomeningeal cells (VLMCs; *Dcn*^+^, cluster 7), ependymocytes (*Elof1*^+^, cluster 8), and endothelial cells (*Itm2a*^+^, cluster 9) (Figure 1D). We found that neurons represented only 9.1% of the total cell population of the ME, of which 79% were GABAergic (Figure S1E).

Interestingly, OPCs (cluster 6) clustered into 2 distinct groups of cells on the tSNE plot (clusters 6a and 6b, Figure 1B), suggesting molecular diversity within this cluster. This finding prompted us to further study this cell population. We extracted all oligodendrocyte lineage cells (clusters 3 and 6 on Figure 1B) and performed a new clustering analysis, revealing 3 cell clusters with distinct molecular signatures (Figures 1E, 1F, and S1F; Table S1).

Established molecular markers for cells in the oligodendrocyte lineage allowed us to characterize cells in cluster 1 as OPCs (express *Pdgfra* and *Cspg4−* correspond to cluster 6a in the main clustering analysis), an immature cell type that differentiates into oligodendrocytes (Marques et al., 2016; Richardson et al., 2011; Figures 1G and 1H). Cells in cluster 2 expressed genes associated with differentiating OPCs (diffOPCs) and newly formed oligodendrocytes such as *Bmp4*, *Fyn*, *Tcf7l2*, and *Enpp6*, which are transcripts previously shown to be specifically expressed in new oligodendrocytes in the adult brain (Marques et al., 2016, McKenzie et al., 2014; Figures 1G and 1H; Figures S1G–S1I). Here, we named these cells diffOPCs (correspond to cluster 6b in the main clustering analysis). We named cells in cluster 3 mature oligodendrocytes (MOLs; correspond to cluster 3 in the main clustering analysis), as they expressed *Opalin*, *Mal*, and *Klk6* (Marques et al., 2016; Figures 1F–1H; Figures S1G–S1I). The distribution of cells expressing stage-specific transcripts indicates that cell coordinates in the tSNE plot reflect cell developmental “age,” following a curve in the plot (Figure 1H; Figure S1I). To confirm how cells in the 3 oligodendrocyte lineage cell clusters are ordered in terms of developmental maturity, we used Monocle (Trapnell et al., 2014) to map cells along a pseudotime axis. This confirmed that defining genes for OPCs, diffOPCs and MOLs are expressed in a sequential order along this axis (Figures S1J–S1L). Thus, the 3 clusters of oligodendrocyte lineage cells represent the successive stages of oligodendrocyte differentiation, from progenitor cells to mature myelinating MOLs.

### diffOPCs and MOLs form a concentrated layer in the dorsal part of the murine and human ME

**Figure 2:**
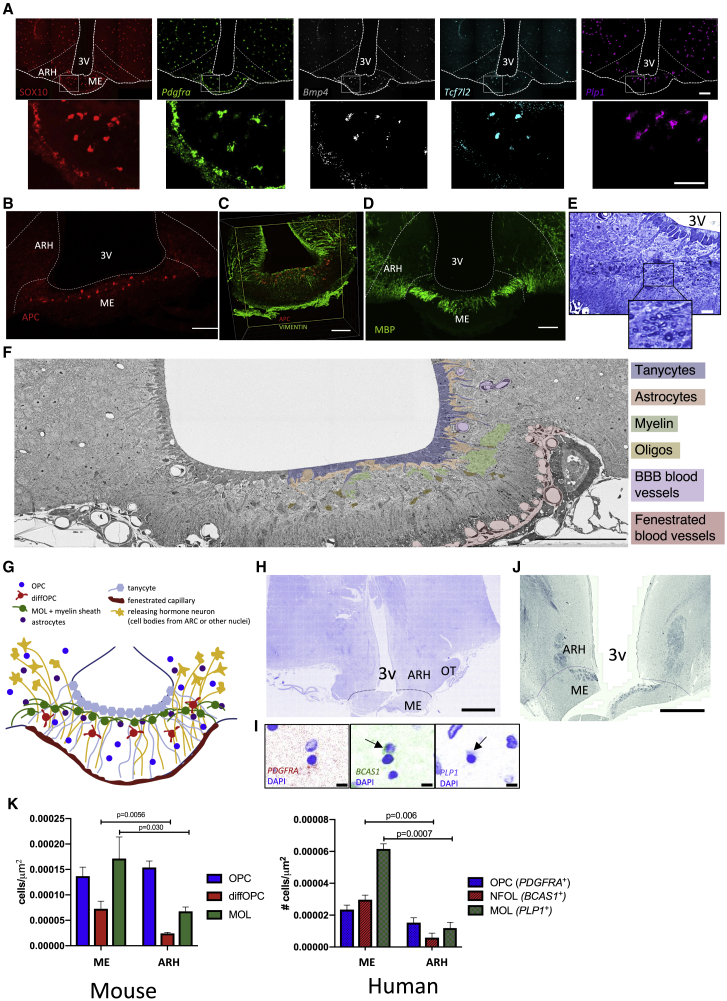
The diffOPC and MOL populations are concentrated in the dorsal part of the murine and human ME (A) FISH combined to immunohistochemistry for detection of oligodendrocyte lineage cells subtype marker gene expression in mouse brain. Scale bar, 100 μm. (B and C) APC expression in thin coronal sections (left; scale bar, 100 μm) (B) and APC and vimentin in thick cleared ME tissue (C). Scale bar, 100 μm. (D) MBP expression in thin coronal sections (left; scale bar, 100 μm) and in thick cleared ME tissue (right). (E) Toluidine-blue-labeled thin ME sections. 3V, third ventricle; scale bar, 10 μm; box indicates inset. Dark circles are transverse cross sections of myelin ensheathing an axon. (F) Block transmission electron micrograph of the ME. Scale bar, 100 μm. (G) Schematic of different cell types of the ME. (H) DAPI stain in human hypothalamus. OT, optic tract; scale bar, 3.75 mm. (I) *PDGFRA*, *BCAS1*, and *PLP1* expression in the human hypothalamus. Arrows indicate probe labeling. n = 2 sections from 1 brain. (J) Luxol fast blue Nissl staining of a human hypothalamic section with ME. Scale bar, 2.5 mm. (K) Measurement of densities of OPCs, diffOPCs and MOLs in the mouse ME and ARH. Error bars depict mean ± SEM (ME, n = 6 animals; ARH, n = 9 animals). (H) Measurement of densities of OPCs (*PDGFRA*^*+*^), NFOLs (BCAS1^+^), and MOLs (*PLP1*^+^) in the human ME and ARH. (n = 1 human, 6 sections; scale bar, 10 μm)

We used RNAscope single-molecule fluorescence *in situ* hybridization (FISH) combined to immunofluorescent labeling to map the density and neuroanatomical distribution of oligodendrocyte lineage subtypes in the murine ME and ARH. We used the corpus callosum (CC) as a reference tissue in which some of the highest levels of new oligodendrocyte formation has been reported in adult mice under physiological conditions (Tripathi et al., 2017; Young et al., 2013; Figures S2A–S2C). We found that OPCs (*Pdgfra*^*+*^/Sox10^+^ cells) are evenly distributed throughout the ME and ARH (Figure 2A), with densities of OPCs in both structures similar to that measured in the CC (Figure S2B). In contrast, the density of diffOPCs (*Bmp4*^+^and/or *Tcf7l2*^*+*^/Sox10^+^) and MOLs (*Plp1*^+^/Sox10^+^/*Bmp4*^*−*^) was more than 3 times higher in the ME than in the ARH (Figures 2A and 2K). Strikingly, diffOPCs and MOLs were almost absent from the ARH and exclusively occupied the dorsal portion of the ME extending bilaterally to the ventral base of the ARH (Figure 2A).

To gain a better understanding of how MOLs are positioned in the ME, we used tissue clearing and immunolabeling for APC (CC1 clone), a marker of MOLs (Bin et al., 2016). Consistent with our findings with FISH, we found that APC^+^ cells exclusively populate the dorsal portion of the ME, immediately below the layer of vimentin^+^ tanycytes (Figures 2B and 2E; Video S1). Labeling of the myelin basic protein (MBP) revealed the presence of dense myelin fibers in the dorsal ME and under the base of the ARH but an absence of myelin in the ARH itself (Figure 2D; Video S2). In line with MBP immunolabeling in thick cleared sections, myelinated axons are seen in the transverse plane, indicating their passage in a rostral-to-caudal direction through the ME (Figures 2E and 2F). In addition, myelinated axons are all localized to the dorsal third of the ME where MBP and *Plp1* are expressed (Figures 2D–2F). Along with knowledge of how neurons and their axons and fenestrated capillaries are positioned in the ME (Langlet et al., 2013a; Yin and Gore, 2010; Figure 2F), we were able to construct a model that showcases the specific and discrete localization of diffOPCs and MOLs in the ME (Figure 2G). Of note, we examined MOL distribution in other circumventricular organs by using APC immunolabeling (Figure S2D). The vascular organ of the lamina terminalis, area postrema, and pituitary appeared mostly devoid from APC^+^ cells.

Video S1. APC and vimentin immunostaining in thick cleared hypothalamic sections, related to Figure 2

Video S2. MBP immunolabeling in thick cleared hypothalamic sections, related to Figure 2

We then used hypothalamic brain sections from an adult human donor to characterize the neuroanatomical distribution of oligodendrocyte lineage cell subtypes in the human MBH and investigate the presence of newly differentiated oligodendrocytes. In the adult human brain, BCAS1 is marker of newly formed oligodendrocytes (NFOLs) (Fard et al., 2017). We used RNAscope to visualize *PDGFRA*, *BCAS1*, and *PLP1* (Figures 2H and 2I) and a luxol fast blue Nissl stain to visualize myelin in the adult human hypothalamus (Figure 2J). These results revealed the presence of NFOLs and thus the continuous formation of new oligodendrocytes in the adult human ME and the discrete localization of myelinated axons in the dorsal human ME. We compared the densities of cells expressing the oligodendrocyte lineage markers in the ME and ARH (Figure 2K). Both in human and mouse ME, we found that OPCs were present at similar densities in ME and ARH, whereas the ME contains 3.5 times more NFOLs/diffOPCs and 4.4 times more MOLs than the ARH (Figure 2K).

### Nutritional signals rapidly regulate the oligodendrocyte transcriptome in the adult ME

We examined the transcriptional signatures of ME cells collected from overnight-fasted and 1-h refed animals. Although such a fast is supraphysiological in the mouse, this paradigm is typically used to identify energy-responsive pathways. Analysis of differentially expressed genes (DEGs) and pathways between fasted and refed mice was used as a hypothesis-generating tool, and *ad-libitum*-fed controls were not included here.

**Figure 3:**
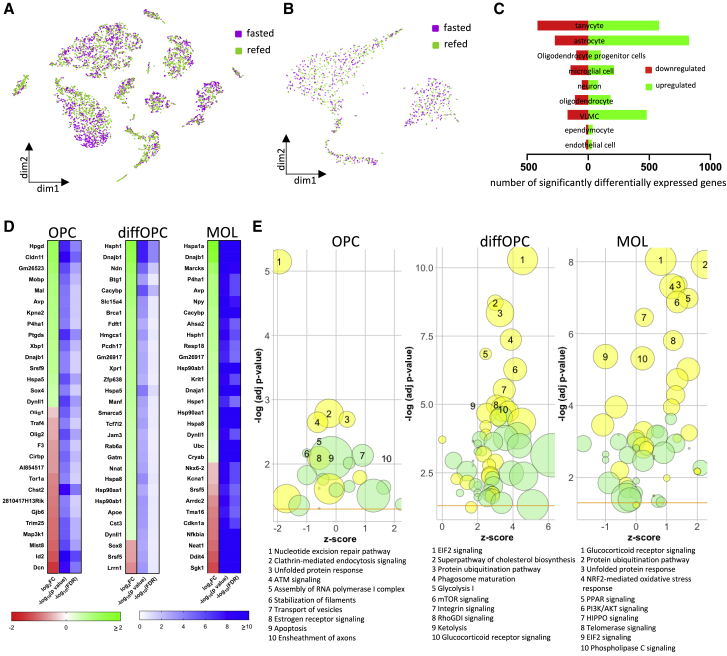
Nutritional signals rapidly regulate the transcriptome of oligodendrocyte lineage cells in the adult ME (A and B) Cell sample treatment group (all ME cells, A; oligodendrocyte lineage cells, B) mapped on tSNE plots (n = 5 per condition). (C) Number of genes significantly different between fasted and refed conditions per cluster (p < 0.05; false discovery rate [FDR], <0.25). (D) Top 30 differentially expressed genes between fasted and refed conditions (p < 0.05; FDR, <0.25; −log_10_(0.05) = 1.3). (E) Graphical representation of top 10 pathways (IPA) changed between fasted and refed conditions. Radius indicates number of differentially expressed genes in current dataset that overlap with IPA gene set; yellow, IPA canonical signaling pathway; green, IPA cellular function; yellow horizontal line, denotes statistical significance threshold (−log(adj pvalue) of 1.3).

Cells from the fasted and refed samples were generally well mixed across all clusters, indicating that fasted and refed conditions do not change gross identities of cells (Figures 3A and 3B). We obtained the list of genes significantly differentially expressed between the refed and the fasted condition in each of the initial 9 cell clusters (Figure 3C; Table S2). Tanycytes, astrocytes, and VLMCs were the most nutritionally responsive cell types, whereas neurons had relatively few DEGs between the fasted and refed conditions. A total of 495 genes were differentially expressed in oligodendrocyte lineage cells, revealing a previously unknown rapid nutritional regulation in this lineage.

To begin to characterize the functional consequences of the fast-refeed stimulus on oligodendrocyte populations, we looked at DEGs in fasted versus refed ME samples in the 3 oligodendrocyte clusters (Figure 3D; Table S2). In all 3 clusters, genes regulating oligodendrocyte lineage progression (OPC migration, proliferation, cell cycle exit and differentiation) represented a large proportion of DEGs, as follows.

In the OPC cluster, genes implicated in OPC migration (*Cldn11* and *Tspan15*) (Tiwari-Woodruff et al., 2001) were upregulated. Genes regulating OPC proliferation were both activated (*Sox4* and *Hes5*) (Braccioli et al., 2018) and inhibited (*Id2* and *Gpr17*) (Sock and Wegner, 2019). Likewise, genes promoting OPC differentiation were both activated (*Kpna2*) (Laitman et al., 2017) and inhibited (*Olig1* and *Olig2*) (Sock and Wegner, 2019). These results suggest that both OPC recruitment and proliferation and OPC differentiation may be upregulated in distinct subsets of OPCs in response to the fast-refeed paradigm. Consistent with a transcriptional activation initiating differentiation into MOLs, refeeding upregulated transcripts related to peroxisomal activity (*Pex5* and *Pmvk*) and involved in cholesterol and myelin lipid synthesis (Bottelbergs et al., 2010; Kassmann, 2014) and myelin-related transcripts (*Mal*, *Mobp*, and *Plp1*). Functional annotation of DEGs in OPCs also highlighted genes involved in hormonal and metabolic sensing (*Mlst8*, *Igf1r*, and *Pik3r1*).

In diffOPCs, only 19 genes were significantly differentially expressed between treatments. Strikingly, they include the transcription factor *Tcf7l2*, a key player in oligodendrocyte differentiation, albeit it has a controversial role on cell cycle exit (Weng et al., 2017). Several transcripts encoding heat shock proteins that promote the survival of myelinating cells were upregulated (*Hsph1*, Dnajb1, *Hspa5*, *Hspa8*, and *Hsp90ab1*) (Zigmond, 2017), as well as genes involved in lipid/cholesterol synthesis or transport (*Hmgcs1* and *Apoe*).

Likewise in MOLs, several transcripts for heat shock proteins were consistently upregulated (*Hspa1a*, *Hsph1*, *Hspa4l*, *Hsp90aa1*, *Hsp90ab1*, *Hspa8*, *Hspa5*, *Cryab*, *Dnaja1*, *Dnajb4*, and *Ahsa2*). Three genes mediating cell cycle exit and differentiation (*Klf6*, *Klf9*, and *Yy1*) (Laitman et al., 2016; Dugas et al., 2012) were upregulated; others were downregulated (*Olig1*, *Olig2*, Sgk1, and Cdkn1a) (Avey et al., 2018). Because all these latter genes are enriched in diffOPCs compared to those in MOLs (Zhang et al., 2014), this finding could indicate lineage progression to MOLs. However, the promyelinating transcription factor *Nkx6-2* (Southwood et al., 2004) and the polarity gene *Mtmr2* required for correct myelin formation (Bolis et al., 2009) were downregulated, whereas genes promoting lipid biogenesis and cholesterol transport (*Elovl1*, *Arid5b*, and *Stard3*) were upregulated, leaving unclear the consequence of refeeding on oligodendrocyte maturation and myelination.

We also used ingenuity pathway analysis (IPA) to make a comprehensive prediction about pathways regulated by the transition from the fasted to refed state in the oligodendrocyte lineage cells of the ME (Figure 3E; Table S3). Consistent with the DEG analysis, top pathways included processes involved in proliferation, cell cycle progression, differentiation, lipid/cholesterol biosynthesis, and myelination (Table S4). In addition, a number of pathways involved in hormonal sensing, growth, and cellular energy handling were significantly regulated (Table S4). This was particularly pronounced in diffOPCs, with a strong activation of EiF2 and mTOR signaling.

Using IPA, we also obtained the top upstream regulators of DEGs, i.e., transcriptional regulators that best explain the expression changes between fasting and refeeding (Table S5). Strikingly, top upstream regulators included two transcription factors necessary for oligodendrocyte differentiation, namely, *Tcf7l2* and myelin regulatory factor (*Myrf*) in all clusters and *Mtor* in OPCs and diffOPCs (Figure S3).

### Nutritional signals rapidly regulate OPC proliferation and differentiation in the ME

**Figure 4:**
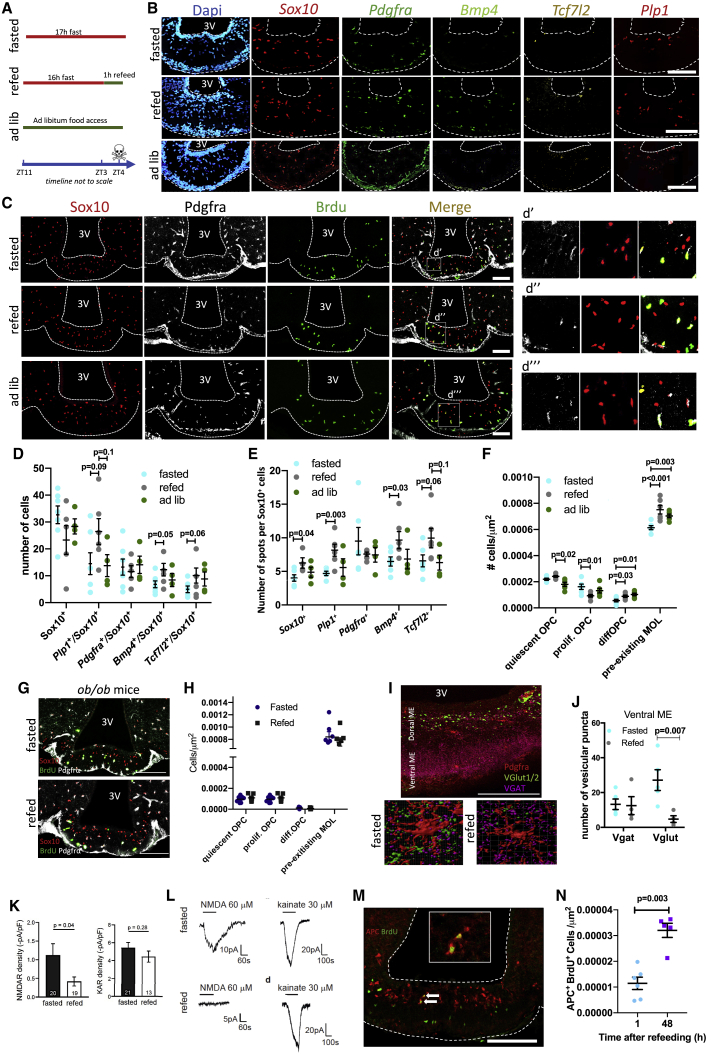
Nutritional signals rapidly regulate OPC proliferation and differentiation in the ME (A) Fast-refeed paradigm used in RNAscope studies. (B) Multiplex single-molecule FISH labeling oligodendrocyte lineage cell markers in the ME (scale bar, 100 μm). (C) BrdU labeling in fasted, refed, and *ad-libitum*-fed mice. Scale bar, 100 μm. (D and E) Number of cells expressing markers (D) and of RNA molecules (“spots”) per cell (averaged per imaged tiles of fixed area) (E) in RNAscope FISH experiment in me sections from fasted (n = 7), 1-h refed (n = 5), or *ad-libitum*-fed (n = 4) mice. (G) Quantification of subsets of BrdU-labeled cells in the ME. Quiescent OPC, Sox10^+^/Pdgfra^+^/BrdU^−^; proliferating OPC, Sox10^+^/Pdgfra^+^/BrdU^+^; diffOPC, Sox10^+^/Pdgfra^−^/BrdU^+^; preexisting MOL, Sox10^+^/Pdgfra^−^/BrdU^−^. (G) BrdU (green) labeling in the ME of fasted and refed *ob/ob* mice and colocalization with Sox10 and Pdgfra. Scale bar, 100 μm. (H) Quantification of subsets of BrdU-labeled cells in the ME of *ob/ob* mice. (I) Colocalization of Pdgfra with Vglut1/2 and Vgat in the ME. Single plane and Imaris 3D reconstruction of z stacks from fasted and refed mice (scale bar, 100 μm). (J) Quantification of vesicular puncta on OPCs in the ventral ME from fasted or refed mice. (K) Density of NMDA receptors (NMDARs) and AMPA/kainate receptors (KARs) in OPCs in fasted and refed mice. The numbers shown on bar graphs represent the number of whole-cell patched ME OPCs. (L) NMDA (60 μM)-evoked and kainate (30 μM)-evoked currents in ME OPCs from fasted or 1-h refed mice. (M) Colocalization of BrdU and APC in ME sections from refed mice 48 h after refeeding (scale bar, 100 μm). (N) Quantification of the density of APC^+^/BrdU^+^ cells in the ME 48 h post-refeeding. Data are means ± SEM.

The analysis of DEGs, top differentially regulated pathways, and top upstream regulators indicates that oligodendrocyte lineage progression is regulated during the fast-refeed transition. We used a number of complementary tools to validate these results and determine how food availability regulates oligodendrocyte lineage progression (Figure 4A). With RNAscope FISH, we found that the expression of *Bmp4* increased with 1-h refeeding compared to that of fasted and/or *ad libitum* states (Figures 4B and 4E), resulting in an increase in the number of diffOPCs (*Sox10*^+^/*Bmp4*^+^) in response to refeeding compared to fasting (Figure 4D). Consistent with an increase in OPC differentiation during the transition from the fasted to the refed state, we observed a trend toward an increase in the expression of *Tcf7l2* in *Sox10*^+^ cells and toward an increase in the number of *Sox10*^+^/*Tcf7l2*^+^ cells (Figures 4B, 4D, and 4E). The number of MOLs (*Sox10*^+^/*Plp1*^+^/*Bmp4*^*−*^) trended toward an increase in refed samples (Figure 4D), and *Plp1* expression, as assessed by the number of Plp1^+^ spots in Plp1^+^/Sox10^+^ cells, significantly increased in the refed versus fasted state (Figure 4E).

To further characterize the effects of nutritional transitions on oligodendrocyte lineage progression, we used an acute BrdU administration protocol and quantified BrdU incorporation into the ME and CC of mice that were either fasted, fasted and refed for 1 h, or maintained with *ad libitum* access to food (Figures S4A and S4B). The total number of cells in the oligodendrocyte lineage (Sox10^+^) did not change between conditions in the ME or CC (Figures S4C and S4D) nor did the number of OPCs (Pdgfrα^+^/Sox10^+^) (Figures S4E and S4F). Cells at different stages of the oligodendrocyte lineage were identified, as follows: non-proliferating/quiescent OPCs (Brdu^−^/Pdgfrα^+^/Sox10^+^), proliferating OPCs (Brdu^+^/Pdgfrα^+^/Sox10^+^), diffOPCs (Brdu^+^/Pdgfrα^−^/Sox10^+^), and pre-exisiting MOLs (Brdu^−^/Pdgfrα^−^/Sox10^+^ and Brdu^−^/APC). BrdU incorporation in oligodendrocyte lineage cells (Brdu^+^/Sox10^+^) was similar between conditions in the CC but trended to be lower in the ME of fasted and refed mice than in *ad-libitum*-fed controls (Figures S4G and S4H), indicating that fasting reduces OPC proliferation. Consistently, there were more non-proliferating OPCs in fasted and refed mice than in *ad libitum* controls (Figures 4C–4F). Refeeding produced a decrease in the number of proliferating OPCs and restored the number of diffOPCs and pre-existing MOLs to *ad libitum* levels (Figures 4C–4F; Figure S4I). Thus, fasting reduces oligodendrocyte lineage progression and 1-h refeeding is sufficient to restore it. None of these changes were observed in the CC, a white matter tract forwhich rapid OPC differentiation has been characterized (McKenzie et al., 2014; Xiao et al., 2018; Figure S4K). In genetically obese and diabetic *ob/ob* mice (Figures S4M and S4N), fasting failed to reduced OPC proliferation, and refeeding did not change the number of diffOPCs (Figures 4G and 4H).

We noticed that diffOPCs were positioned more dorsal in the ME of refed mice than in fasted controls, suggesting that refeeding also promotes the dorsal migration of OPCs committed to differentiation (Figures S4L–S4O). Consistent with the idea that activated OPCs ready to be differentiated are migrating dorsally, we found that dorsal OPCs receive significantly less synaptic input than ventral OPCs, a hallmark of OPC differentiation (Spitzer et al., 2016), and that refeeding decreased the number of glutamatergic puncta contacting ventral OPCs (Figures 4I and 4J; Figure S4P). We further explored the electrophysiological changes in ME OPCs in fasted and refed mice by using *NG2-EYFP:FUCCI2a* mice in which all OPCs are labeled with EYFP and mCherry is expressed during the G_0_/G1 phase (Mort et al., 2014). With this model, OPCs in the active cell cycle appear green (Fucci-mCherry^−^/EYFP^+^), whereas quiescent OPCs appear yellow-green (Fucci-mCherry^−^/EYFP^+^). Both during live imaging and in fixed-stained sections, we found an increased number of cycling OPCs in ME sections from refed mice compared with that in fasted controls (Figures S4Q and S4R). Electrophysiological assessments revealed that the density of NMDA receptors (NMDARs) in ME OPCs decreased significantly in refed mice and NMDA-evoked currents were significantly blunted (Figures 4K and 4L). In contrast, the density of AMPA/kainate receptors (KARs) and kainate-evoked currents remained unchanged between conditions (Figures 4K and 4L). These data provide additional support for the conclusion that refeeding increases OPC differentiation in the ME.

Last, we examined the fate of recently differentiated OPCs in the fasting-refeeding paradigm. We determined if recently divided cells (Brdu^+^) expressed markers of MOLs in the time frame of the 1-h refeeding period and after 48 h and 72 h by costaining for BrdU and APC (a marker of MOLs) in the sections of mice exposed to the acute BrdU exposure paradigm (Figure S4A). The density of BrdU^+^ and APC^+^ cells remained stable at 48 h and 72 h after refeeding (Figure S4S). Fasting reduced the number of APC^+^ cells in the ME and 1-h refeeding was sufficient to restore *ad libitum* levels (Figures S4T and S4U). The 1-h refeeding period produced a trend toward an increase in the number of Brdu^+^/APC^+^ cells (Figure S4V), which further increased after 48 h, at a 3-fold higher number than that after 1-h refeeding (Figures 4M and 4N) with no further increase at 72 h (not shown), indicating newly differentiated OPCs survive and become MOLs.

Previous reports indicate the possibility of very rapid changes in myelination (within hours) in zebrafish and humans (Czopka et al., 2013; Hofstetter et al., 2013). Many genes translating to proteins involved in myelination are regulated by 1-h refeeding in the ME (Table S4; Figure S4W), prompting us to examine whether increased oligodendrocyte differentiation after refeeding was associated with changes in ME myelination. We used toluidine blue labeling of thin sections to view the distribution of myelinated axons in the ME and transmission electron microscopy to quantify the density of myelinated axons and myelin thickness in the ME of fasted and refed mice (Figure S4X). We did not detect any changes in these metrics (Figures S4Y–S4Aa), indicating that in this time frame, changes in myelination do not occur.

Collectively, these data indicate that refeeding after an overnight fast rapidly promotes the formation of new oligodendrocytes and demonstrate the unique ability of oligodendrocyte lineage cells of the ME to respond to nutritional stimuli.

### Nutritional regulation of oligodendrocyte lineage progression in the ME regulates local PNNs

We then characterized the functional consequences of the rapid nutritional regulation of oligodendrocyte lineage cells in the ME. We focused on DEGs encoding secreted proteins that alter the local ME environment and are potential modulators of sensing properties of local neurons and tanycytes. Strikingly, a number of genes expressing proteins of the extracellular matrix (ECM) were among the top DEGs in oligodendrocyte lineage cells (Table S3). Of note, oligodendrocyte lineage cells are the sole or main producers of key components of PNNs, including tenascin-R (*Tnr*), versican (*Vcan*), and phosphacan (*Ptprz1*), as well as enzymes involved in proteoglycan formation, modification, or degradation (*Adamts4*, *Chst2*, *Chst4*, and *Chst5*) (Figures S5A–S5F; Lemarchant et al., 2013; Miyata and Kitagawa, 2016, 2017; Narentuya et al., 2019). Recently, PNNs have been shown to enmesh key metabolic sensing neurons at the ARH-ME junction and have been proposed to regulate how these neurons access blood-borne signals (Mirzadeh et al., 2019). Thus, we reasoned that the rapid changes in oligodendrocyte lineage cells in response to fasting-refeeding may produce changes in the ME ECM composition, including changes in PNNs, leading to changes in ARH-ME metabolic sensing.

**Figure 5:**
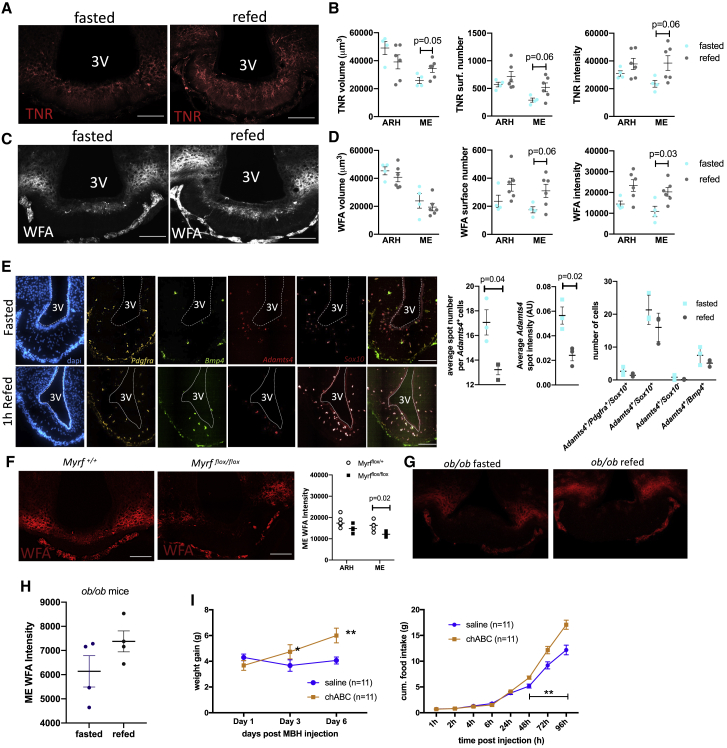
Nutritional regulation of oligodendrocyte lineage progression in the ME regulates local perineuronal nets (A–D) Maximum projection stacks of 20 μM ME sections immunolabeled against TNR and WFA (A and B) and volumetric, surface number, and intensity quantification in these stacks (C and D). Scale bar: 100 μm. (E) Multiplex single-molecular FISH labeling of oligodendrocyte lineage cell markers and *Adamts4* in the ME of fasted and refed mice. Scale bar, 100 μm. (G) WFA immunolabeling in the ME of mice with adult deletion of *Myrf* in OPCs (*Myrf*^*flox/flox*^) and controls (*Myrf*^*+/+*^). Scale bar, 100 μm. (G and H) WFA immunolabeling in the ME of *ob/ob* mice after an overnight fasted or an overnight fast followed by a 1-h refeed. (I) Weight gain and cumulative food intake in mice treated with a local MBH injection of chABC or vehicle (saline). Data are means ± SEM.

To test this hypothesis, we performed immunolabeling against tenascin R (TNR), an ECM glycoprotein that promotes the assembly of PNNs (Morawski et al., 2014) and is highly enriched in diffOPCs (Figure S5B), to test the prediction that an increased number of diffOPCs following a refeed may increase TNR expression in the ME. We also stained for Wisteria floribunda agglutinin (WFA), a lectin that binds to the glycosaminoglycan (GAG) chain of chondroitin sulfate proteoglycans and is routinely used to detect PNNs. In line with the increased number of diffOPCs, we found higher TNR immunolabeling in the ME of refed mice (Figures 5A and 5B). This finding was accompanied by an increase in WFA immunolabeling in the ME of refed mice, supporting an acute nutritional regulation of WFA^+^ ECM structures in this paradigm (Figures 5C and 5D).

*Adamts4* (disintegrin and metalloproteinase with thrombospondin motifs 4) drew our attention as an additional potential mediator of the nutrition-induced changes in PNN density in the ME. *Adamts4* can cleave large chondroitin sulfate hyaluronan-binding proteoglycans (CSPGs), core components of PNNs (Bozzelli et al., 2018). It is specifically expressed in oligodendrocyte lineage cells (Pruvost et al., 2017) and among the top DEGs significantly downregulated in refed samples compared to fasted samples (Figure 3D; Table S3). Thus, increased expression of *Adamts4* during fasting may contribute to the rapid degradation of PNN in the ME-ARH region. To confirm this idea, we quantified the expression of *Adamts4* in ME oligodendrocyte lineage cells from fasted and refed mice by using RNAscope. All used metrics indicate an increased *Adamts4* expression in oligodendrocyte lineage cells in the ME of fasted mice (Figure 5E).

To directly test the role of OPC differentiation in the changes in density of the WFA^+^ ECM structure observed during the fasting-refeeding transition, we used a mouse line carrying a floxed allele of *Myrf*, which is required for the OPC differentiation, on a *Pdgfra-CreERT2: Rosa-YFP* background to obtain *Myrf*^*(+/+)*^ and *Myrf*^*(flox/flox)*^ (*Myrf* KO) mice (McKenzie et al., 2014). Administration of tamoxifen in adults induces Cre-mediated inactivation of *Myrf* in *Pdgfra*-expressing OPCs while simultaneously labeling OPCs with yellow fluorescent protein (YFP). At 3 weeks post-tamoxifen administration, we confirmed a significant reduction of OPC differentiation in *Myrf* KO mice (Figure S5G). Strikingly, deletion of *Myrf* in OPCs dramatically reduced WFA immunoreactivity in the ME (Figure 5f), indicating that OPC differentiation is required for WFA^+^ ECM structure assembly in the ME. In *Myrf* KO mice, refeeding failed to increase WFA immunolabeling in the ME (Figure S5H), supporting the conclusion that OPC differentiation is necessary for the nutritional control of ME ECM remodeling. Consistently, in *ob/ob* mice (Figures S4M and S4N) in which the nutritional control of oligodendrocyte lineage progression is blunted, ME WFA immunoreactivity is low and fails to respond to refeeding (Figures 5G and 5H).

Last, we assessed the functional consequences of ECM disruption in the ARH/ME region by performing local injections of chondroitinase ABC (chABC) to acutely digest the ECM GAG chains in this region in wild-type (WT) mice (Alonge et al., 2020). As expected, chABC injection produced a significant reduction in WFA immunolabeling in the ARH-ME region (Figure S5I). During the first 2 days post-injection, no changes in food intake and body weight were detected. However, mice treated with chABC started to eat more than controls starting 3 days after the injection, producing a significant increase in weight gain that was maintained for the 3 following days (Figure 5I). Thus, assembly of chondroitin sulfate proteoglycans in the ME-ARH region regulates energy balance.

### mTORC1 is highly active in oligodendrocytes in the ME and responds to nutritional signals

**Figure 6:**
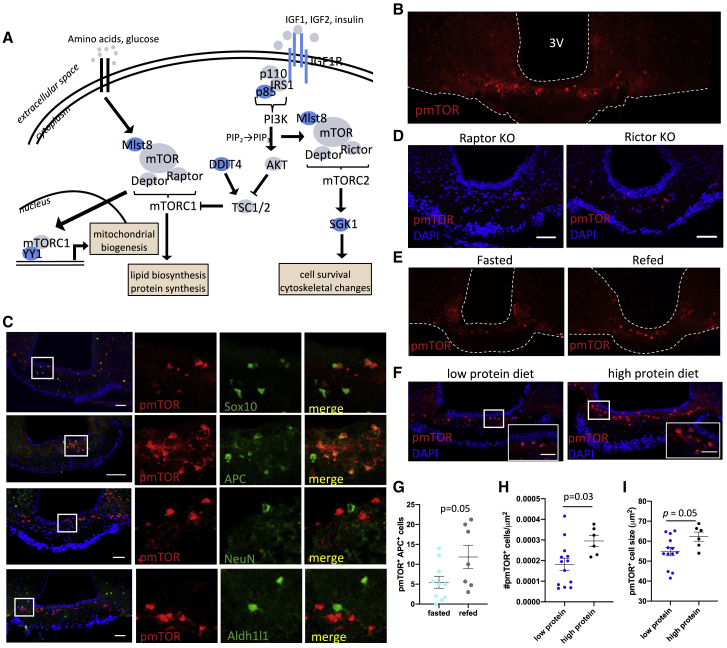
mTORC1 signaling is highly active and nutritionally regulated in the ME oligodendrocytes (A) Schematic of mTOR signaling pathway with DEGs labeled in blue. (B) Immunolabeling for phosphorylated mTOR (pmTOR) in the ME. Scale bar, 100 μm. (C) ME colocalization of pmTOR with Sox10, APC, NeuN, or Aldh1l1. Scale bars, 100 μm. (D) pmTOR labeling in ME tissue from oligodendrocyte-specific raptor KO or rictor KO. Scale bars, 100 μm. (E and G) pmTOR immunolabeling in the ME of mice fasted overnight or refed for 1 h. Scale bar, 100 μm. (G–I) pmTOR immunolabeling in the ME of mice fed a low or high protein diet. Scale bars, 25 μm. Data are mean ± SEM.

To follow up on the IPA results highlighting a role for mTOR in the rapid nutritional regulation occurring in ME oligodendrocyte lineage cells, we further investigated the nutritional regulation of this pathway. Consistent with IPA, several genes involved in the mTOR signaling pathway are significantly regulated by the refeeding stimulus (Figure 6A; Figure S6A). mTOR signaling plays a key role in OPC differentiation, oligodendrocyte maturation, and myelination (Kim and Guan, 2019) and is, ubiquitously, a key cellular integrator of nutritional and hormonal signals in the regulation of many anabolic processes (Laplante and Sabatini, 2009). Thus, mTOR signaling in oligodendrocytes may couple metabolic sensing to changing in OPC differentiation and/or MOL maturation and myelination. Of note, OPCs, diffOPCs, and MOLs express genes to receptors for many of the metabolic hormones that signal energy availability to the hypothalamus, including Adipor2, Fgfr2, Inr, Lepr, Ghr, Gipr, and Thra (Figure S6B), of which many have been shown to activate hypothalamic mTOR signaling (Bibollet-Bahena and Almazan, 2009; Cui et al., 2010; McKinnon et al., 1993; Talbott et al., 2007).

We first assessed the expression of the active form of mTOR in the mouse ME by using an antibody detecting mTOR phosphorylated at Ser2448 (pmTOR). pmTOR immunolabeling in the ME is remarkably bright in a cell population lying in the dorsal ME (Figure 6B). Similar labeling of pmTOR was absent from the rest of the brain, including other circumventricular organs (Figure S6C). To confirm the identity of the cells expressing pmTOR, we colocalized pmTOR with established markers of oligodendrocytes, neurons, and astrocytes. ME pmTOR immunolabeling was absent from neurons and astrocytes but colocalized with Sox10, which is expressed across the oligodendrocyte lineage (Figure 6C). A total of 37.3% ± 4.54% of Sox10^+^ cells were pmTOR^+^, showing that only a subset of oligodendrocyte lineage cells of the ME have active mTOR signaling. In fact, the ME pmTOR^+^ signal highly colocalized with the postmitotic oligodendrocyte marker APC (Figure 6C), indicating that newly differentiated oligodendrocytes or MOLs and not OPCs have active mTOR signaling.

mTOR can signal by two different complexes, resulting in the activation of distinct transcriptional programs including protein/lipid synthesis or cytoskeletal organization (Laplante and Sabatini, 2012). To investigate if mTOR signaling was occurring by the mTOR Complex 1 (mTORC1; contains raptor protein) or Complex 2 (mTORC2; contains rictor protein), we took advantage of exclusive expression of 2′,3′-cyclic-nucleotide 3′-phosphodiesterase (CNPase) in oligodendrocytes and used CNP-Cre:raptor^fl/fl^ (raptor knockout) and CNP-Cre:rictor^fl/fl^ (rictor KO) mice to interrogate which signaling complex is most active in oligodendrocytes of the ME. pmTOR labeling of tissues from raptor or rictor KO mice revealed the absence of labeling in raptor KO mice (Figure 6D), thus indicating that mTORC1 signaling is highly active in ME oligodendrocytes.

To validate the result from analysis of our scRNA-seq dataset indicating that mTOR signaling is regulated by fasting and refeeding in ME oligodendrocytes, we tested whether mTOR activity in ME oligodendrocytes would change in this paradigm. Refeeding significantly increased the number of pmTOR^+^ oligodendrocytes (Figures 6E and 6G). In addition, there was a trend for an increase in cell size in pmTOR^+^ oligodendrocytes (p = 0.08) and increased signal intensity (p = 0.07). However, when refed mice were pretreated with the mTORC1 inhibitor rapamycin, OPC proliferation and differentiation were unaffected (Figure S6D), suggesting that increased mTORC1 signaling in oligodendrocytes in response to refeeding may instead initiate an increase in oligodendrocyte maturation.

Many nutritional and hormonal signals in the refed state could potentially produce increased mTORC1 activity in oligodendrocytes. To further test the idea that nutritional signals are sensed by mTORC1 in oligodendrocytes to promote differentiation, we assessed the effect dietary proteins on OPC differentiation. This choice was based on the fact that dietary protein is one of the strongest nutritional signals that activates mTORC1 signaling in multiple tissues. In addition, *Bckdk*, which encodes one of the key enzymes in branched-chain amino acid metabolism, is one of the top upstream regulators explaining the transcriptional changes during the fast refeed transition in MOLs (Figure S3). Mice were exposed to a low or high protein diet for 4 days, a time frame during which these diets do not differentially impact weight gain (Figure S6E). We found that high protein feeding increased the density of pmTOR^+^ oligodendrocytes and increased the size of pmTOR^+^ cells (Figures 6F, 6H, and 6I). However, this was not associated with a change in OPC proliferation and oligodendrocyte lineage progression, as assessed with Brdu incorporation during the last 24 h of the dietary paradigm (Figure S6F).

## Discussion

Our study provides a high-resolution transcriptional characterization of distinct cell types of the ME and their response to nutritional transitions, allowing the identification of cell-type-specific cellular processes regulated by nutrient availability. This characterization represents a useful resource for the community. Among other uses, it may help understand the molecular specificities of cells directly exposed to variations in circulating signals and be well positioned to be involved in homeostatic nutritional and metabolic interoception. The tanycytic and astrocytic populations appear relatively homogeneous molecularly, but how they may differ from parenchymal populations remains to be determined.

One striking observation was the presence of 3 subtypes of oligodendrocyte lineage cells, in particular intermediate premyelinating oligodendrocytes in the murine and human ME and in high proportions of the total pool of oligodendrocyte lineage cells. Remarkably, we found that acute changes in energy availability are sufficient to produce a robust transcriptional response in oligodendrocyte lineage cells, leading to changes in OPC proliferation, production of new oligodendrocytes, and oligodendrocyte maturation, as well as changes in the number of existing MOLs. The main effect of the fast as compared to the *ad-libitum*-fed condition is a reduction in the number of existing MOLs. In contrast, refeeding triggers changes throughout the lineage. Although it is clear that OPCs change state with refeeding, expression of *Bmp4* and loss of *Pdgra* are not sufficient to conclude that they undergo full differentiation. Nevertheless, other features including OPC migration and changes in electrophysiological properties, as well as increased number of APC^+^ oligodendrocytes after the refeed, support the conclusion that refeeding promotes oligodendrocyte differentiation. Consistent with this conclusion, we observed high mTORC1 activity in ME oligodendrocytes after refeeding and increased oligodendrocyte cell size, which is typical of cells preparing for myelination. Oligodendrocytes derived from recently differentiated OPCs (Brdu^+^/APC^+^) survived to contribute to the ME. Intriguingly, the number of APC^+^ cells returned rapidly to baseline levels after the refeed, suggesting that refeeding may represent a homeostatic response to restore the MOL population compromised during the fast. Oligodendrocytes are normally long lived, and adult-born oligodendrocytes add myelin to existing structures (Hughes et al., 2018; Tripathi et al., 2017). Thus, further studies are needed to characterize the stability of ME oligodendrocytes and the longer-term fate of adult-born oligodendrocytes in the ME.

The lack of changes in myelination in the time frame of the fast-refeed paradigm suggest that acutely, oligodendrocyte plasticity may contribute to hypothalamic functions through the regulation of the transient intermediate-stage population. One functional myelin-unrelated consequence that we identified is the regulation of PNN assembly and remodeling. PNNs are emerging as important components of the cytological architecture of the ME-ARH barrier that is critically involved in the central control of energy balance in obesity and type 2 diabetes models (Alonge et al., 2020; Mirzadeh et al., 2019). We show that the density of ME PNNs is reduced significantly after a fast and restored with a 1-h refeed. Nutritional regulation of ME PNNs is blunted in mice with induced deletion of *Myrf* in OPCs, directly supporting a role of OPC differentiation in the nutritional regulation of ME PNNs. We propose 2 candidate mechanisms through which OPC differentiation may alter PNN stability, as follows: increased production of PNN-stabilizing protein TNR by diffOPCs in the refed state and increased production of PNN-dismantling enzyme Adamts4 in the fasted state. These results extend the role of ME PNNs to include the homeostatic control of feeding in lean mice and identify PNNs as contributors to ME structural plasticity in response to nutritional cues.

Given the reduction in the number of MOLs in response to fasting, it is tempting to speculate on potential myelin-related functional consequences in a longer time frame. Myelin axons passing through the ME are axons from oxytocin and vasopressin neurons (Yin and Gore, 2010) that release hormones to the pituitary to regulate parturition/lactation and blood pressure/diuresis, respectively (Gimpl and Fahrenholz, 2001, Koshimizu et al., 2012). To our knowledge, myelin plasticity on these axons has not been described. Although fasting-refeeding do not produce changes in pituitary oxytocin and vasopressin content (Burlet et al., 1992), further studies are needed to clarify this question.

Our results provide knowledge on the regulation of oligodendrocyte lineage cells by nutritional signals, raising the question of how oligodendrocytes of other brain sites respond to blood-borne signals when the BBB is compromised, such as after brain injury. Interestingly, in this pathological context, both increased oligodendrocyte production and remodeling of the ECM are occurring (Edwards and Bix, 2019). Whether remyelination post-injury is regulated by peripheral energy availability has not been determined to our knowledge. Coupling oligodendrocyte differentiation to energy availability seems appropriate, given the high energetic cost of myelination and requirement for specific nutrients. Extensions of our findings to these models may support future nutrition-based strategies to optimize remyelination after injury and better understand the regulatory role of the nutritional and metabolic status on oligodendrocyte biology.

Our data support a role for mTORC1 in oligodendrocytes as a signaling pathway activated by nutrient availability. mTORC1 activation could occur in response to direct sensing of extracellular nutrients and/or secondary to changes in local neuronal activity in response to refeeding (Saxton and Sabatini, 2017). The regulation mTORC1 signaling specifically in oligodendrocytes by dietary proteins indicates that direct sensing of circulating amino acids can mimic some of the consequences of refeeding on oligodendrocyte lineage cells. The lack of an effect of rapamycin on OPC proliferation and differentiation suggests that mTORC1 is involved in downstream events, i.e., preparation for myelination as discussed above. The remarkably unique high level of mTORC1 expression and mTORC1 nutritional regulation in ME oligodendrocytes are likely a consequence of the exclusive environment of the ME with unbuffered access to peripheral nutrients and metabolic signals. However, similar expression and regulation were not observed in other circumventricular organs containing oligodendrocytes, suggesting that other aspects specific to the ME niche are also involved.

## STAR★Methods

### Key resources table

**Table:** 

REAGENT or RESOURCE	SOURCE	IDENTIFIER
**Antibodies**		

donkey anti-chicken Alexa Fluor® 488-conjugate	Jackson Immunoresearch	Cat# 703-545-155; RRID:AB_2340375
donkey anti-goat Alexa Fluor® 488-conjugate	Thermofisher	Cat# A11055; RRID:AB_2534102
donkey anti-goat Alexa Fluor® 594-conjugate	Thermofisher	Cat# A11058; RRID:AB_2534105
donkey anti-mouse Alexa Fluor® 488-conjugate	Thermofisher	A Cat# 21202; RRID:AB_141607
goat anti-mouse CF640R	Sigma	Cat# SAB4600346; RRID:AB_10853309
Streptavidin 594 Alexa Fluor	Life Technologies	Cat# S32356
donkey anti-rabbit Alexa Fluor® 488-conjugate	Thermofisher	Cat# A21206; RRID:AB_2535792
donkey anti-rabbit Alexa Fluor® 594-conjugate	Thermofisher	Cat# A21207; RRID:AB_141637
donkey anti-rat Alexa Fluor® 488-conjugate	Thermofisher	Cat# A21208
goat anti-guinea pig Alexa Fluor 594	Thermofisher	Cat# A11073; RRID:AB_2534117
APC (clone CC1)	Millipore	Cat# OP80; RRID:AB_2057371
Brdu	biorad	Cat# OBT-0030; RRID:AB_2341179
GFP	Abcam	Cat# Ab13970; RRID:AB_300798
MBP	Abcam	Cat# ab7349; RRID:AB_305869
NeuN (clone A60)	Millipore	Cat# MAB377; RRID:AB_2298772
Pdgfra	Cell Signaling Technology	Cat# 3164; RRID:AB_2162351
pmTOR (Ser 2448, clone 49F9)p	pmTOR (Ser 2448, clone 49F9)	Cat# 2976; RRID:AB_490932
Olig2	Millipore	Cat# MABN50; RRID:AB_10807410
Sox10 (to human)	R&D Systems	Cat# AF2864; RRID:AB_442208
Tnr	Synaptic Systems	Cat# 217 011; RRID:AB_2256347
Vgat	Synaptic Systems	Cat# 131 004; RRID:AB_887873
Vglut1/2	Synaptic Systems	Cat# 135304; RRID:AB_887878
Vimentin	Millipore	Cat# ab5733; RRID:AB_11212377
WFA	Vector Biolabs	Cat# B-1355; RRID:AB_2336874

**Chemicals, peptides, and recombinant proteins**		

Brdu	Sigma	Cat# 59-14-3
chABC	Sigma	Cat# 9024-13-9
Neurobasal A	Thermofisher Scientific	Cat# 10888022
papain	Worhtington Biomedicals	Cat# LS003118
DNase I	Sigma	Cat# D4263
CUBIC1 solution	VWR Chemical	Cat# 443874G
Quadrol	Sigma	Cat# 122262

**Experimental models: Organisms/strains**		

C57BL/6j	Charles River	N/A
*CNP-Cre:raptor*^*fl/fl*^	A gift from Macklin Lab, University of Denver	N/A
*CNP-Cre:rictor*^*fl/fl*^	A gift from Macklin Lab, University of Denver	N/A
*Aldh1l1-GFP*	A gift from Tschop Lab, Technische Universität München	N/A
*NG2-EYFP:FUCCI2a*	A gift from Karadottir Lab, Cambridge Stem Cell Institute	N/A
*Myrf*^*flox/flox*^	Jackson	Cat# 010607
*Pdgfra-CreERT2*	A gift from Richardson Lab, University College London	MGI 3832569
*ob/ob*	Jackson	Cat# 000632

**Software and algorithms**		

Ingenuity PAthways Analysis	QIAGEN	https://digitalinsights.qiagen.com/products-overview/discovery-insights-portfolio/analysis-and-visualization/qiagen-ipa/
Imaris	Oxford Intruments	https://imaris.oxinst.com/microscopy-imaging-software-free-trial
Harmony	Perkin Elmer	https://www.perkinelmer.com:443/product/harmony-4-9-office-license-hh17000010
R	R Project	https://www.r-project.org/

**Deposited data**		

scRNaseq data	GEO	GSE133890

### Resource availability

#### Lead contact

Further information and requests for resources and reagents should be directed to and will be fulfilled by the Lead Contact, Clemence Blouet (csb69@medschl.cam.ac.uk).

#### Materials availability

This study did not generate any new unique reagent.

#### Data and code availability

All code used for R analysis of scRNaseq data is found in the online repository Github: Kohnke-et-al-2019 _publish (version of record available at https://zenodo.org/record/4893941). scRNaseq data are available on GEO (GEO: GSE133890).

### Experimental model and subject details

#### Animals

8-9-week-old male C57BL/6j mice were used in all studies except where otherwise noted. Mice were maintained on a 12h light/dark cycle, had free access to water and normal chow (Safe Diets – Safe 105 m) and were group-housed (at least 2 animals per cage). Animals were handled regularly before experiments to reduce stress-related responses. All studies were approved by the local Ethics Committee and animals were treated in accordance with the UK Home Office (Scientific Procedures) Act (1986). *CNP-Cre:raptor*^*fl/fl*^ and *CNP-Cre:rictor*^*fl/fl*^ brain tissue was provided by Professor Macklin from the University of Colorado Anschutz Medical Campus. *Aldh1l1-GFP* mice were provided by Professor Tchöp from the Helmholtz Diabetes Center & German Center for Diabetes Research.

Other mouse strains used in this manuscript are: *NG2-EYFP:FUCCI2a* Fucci mice (IMSR Cat# RBRC06511), *Pdgfra-CreERT2* (MGI 3832569), *Myrf* ^*flox/flox*^ (Jax 010607) and *ob/ob* mice (Jackson Labs, 000632).

#### Humans

Human hypothalamic tissues used for this study were from male donors to the Cambridge Brain Bank. Donors gave informed written consent for the use of brain tissue for research and tissues obtained were used in accordance with the Research Ethics Committee Approval number 10/H0308/56. Samples were from an 83-year-old with no neuropathology.

### Method details

#### Fast-refeed paradigm

All mice were maintained group housed as we observed in pilot studies that the stress induced by single housing produced variability in the outcome measures. Food was removed just before dark onset (ZT11) for 16h and access to food was restored at ZT3 in refed animals for 1h before sacrifice at ZT4. Visual inspection of the stomach’s content after sacrifice allowed us to conform that all animals from the refed group ate comparable amounts of food. In studies where we examined refeeding-induced changes in myelin, we used the same paradigm but a refeeding period of 2h to increase our chances of seeing altered myelin thickness. For the BrdU labeling experiment only, animals were fasted for 24h beginning at noon (ZT6) to allow the administration of 4 ip BrdU doses (50mg/kg) during the fast.

#### Low protein/high protein paradigm

After 3 consecutive brief exposures to the novel diets to avoid neophobia, mice were fed isocaloric diets containing either 7% or 45% of energy as casein for 4 days (Research diets, LP: D17030701, HP: D17030703, respectively). The LP diet consisted of 20% kcal fat (from soybean oil), 73% kcal carbohydrate (from corn starch and sucrose), and 7% kcal protein (from casein). The HP diet consisted of 20% kcal fat (from soybean oil), 35% kcal carbohydrate (from corn starch and sucrose), and 45% kcal protein (from casein).

#### Bromodeoxyuridine (BrdU) administration for fast-refeed experiment

In these experiments, mice were fasted for 24h to allow the administration of 2 ip Brdu doses per day and sufficient labeling of proliferating cells, as follows. Mice were fasted at noon and received 2 ip injections of BrdU (Sigma, 50 mg/kg in saline), at ZT7 and ZT11 respectively. The next day they received 2 additional BrdU injections at ZT3 and ZT5, followed by a refeed of 1h or an additional 1h of fast, and sacrifice at ZT7. For the mTORC1 inhibition experiment, mice were fasted at ZT6, administered with 4 ip Brdu doses as above, refed at ZT6 for 1h and were treated with 10mg/kg rapamycin ip. prepared in 10% DMSO at the beginning of the refeeding period.

#### ChABC injections

Surgical procedures were performed under isofluorane anesthesia, and all animals received Metacam prior to the surgery, 24 hr after surgery and were allowed a 1-week recovery period during which they were acclimatized to injection procedures. Mice were stereotactically implanted with bilateral steel guide cannulae (Plastics One) positioned 1 mm above the ME-ARH region (A/P: −1.1 mm, D/V: −4.9 mm, lateral: +0.4 mm from Bregma), as previously described (Blouet et al., 2012). Cannula guides were secured in place with Loctite glue and dental cement (Fujicem). 10 days post-surgery, bevelled stainless steel injectors (33 gauge) extending 1 mm from the tip of the guide were used for injections of chABC (Sigma, 20mU in 50 nL bilaterally) or vehicle. Correct targeting was confirmed histologically postmortem (placement of cannula guide track).

#### Perfusion fixation

Animals were anaesthetized with an ip injection of 50 ul pentobarbitol (Dolethal, 200 mg/ml) then transcardially perfused as follows. For immunohistochemistry (IHC), tissue clearing, and RNAscope experiments, animals were perfused with 0.01 M phosphate buffered saline (PBS) at room temperature (RT) followed by 4% paraformaldehyde (PFA, Fisher Scientific) in PBS (pH 7.4) at 4°C. For experiments requiring resin embedding, animals were perfused with cold 4% glutaraldehyde (Generon), 0.008% CaCl2 (Sigma) in PBS.

#### Tissue dissection and dissociation

Tissue dissociation for single-cell RNA sequencing (scRNaseq) was performed as previously described (Lam et al., 2017). 10 P40-47 mice were fasted overnight, half were refed for 1h as described above. Animals were sacrificed via cervical dislocation and the brain was quickly extracted into cold Neurobasal-A medium (ThermoFisher Scientific). The ME was dissected from each brain with fine curved scissors (Fine Science Tools - No. 15010-11). MEs were placed in ice cold papain (Worthington Biochemicals - LK003160, 20 U/ml in Hibernate A) in separate 1.5 mL Eppendorfs until all dissections were finished. The sections in papain were incubated at 37°C (500 rpm) for 15-20 min, and the Eppendorfs were swirled every 5 min. The tissue was then triturated in prewarmed DNase I solution (Sigma-Aldrich - D4263) at 37°C then placed in tubes on ice until fluorescence-activated cell sorting (FACS).

#### Fluorescence-activated cell sorting

The cell suspensions from triturations were passed through a 40 um cell strainer into fresh collection tubes. DraQ5 and DAPI were added to the samples in DNase solution to select for nuclei and exclude dead cells, respectively, then single cells were sorted with an Influx Cell Sorter (BD Biosciences) into tubes containing 10 ul 0.4% BSA in Ca-/Mg-free PBS. 3500 cells were sorted into each tube then kept on ice until sequencing.

#### Sequencing

Isolated cells were encapsulated in droplets and cDNA libraries were made using a 10X Genomics Chromium instrument and 10X Single Cell 3′ V2 Reagent kit. Paired end sequencing was performed on an Illumina HiSeq 4000. The first 26bp read contains both a cell barcode and a unique molecular identifier, the next 76bp read contains the cDNA insert. The sequencing reads were mapped to the Genome Reference Consortium m38 (mm10) mouse reference genome and counted using 10X Genomics Cell Ranger software version 2.0. Library preparation and sequencing was performed at the Genomics Core, Cancer Research UK Cambridge Institute. Sequencing data are available on GEO (accession number GSE133890). Of 14,000 cells captured via FACS, 5,982 cells were successfully sequenced using the 10X platform Single Cell 3′ v2. A median of 1,853 genes were expressed per cell, with a median of 3,837 UMI counts per cell.

#### T-distributed stochastic neighbor embedding and clustering

R software was used to analyze scRNaseq data. The R package ‘cellrangerRkit’ (supported by 10X Genomics) was used to perform t-distributed stochastic neighbor embedding (TSNE), a dimensionality reduction technique that allows mapping of all cells in 2 dimensions based on their transcriptomic profile. The package ‘NBClust’ (Lam et al., 2017) was used to test the TSNE plot for the optimal number of clusters. Finally, cellrangerRrkit was used to find the top defining genes per cluster.

#### Differential gene expression

The R package ‘edgeR’(McCarthy et al., 2012) was used to identify differentially expressed genes (DEGs) between fasted and refed conditions in individual clusters. This package fits gene expression data from one cluster from one condition to a generalized linear model (GLM) then compares it to expression in the same cluster in the other condition. GLM fitting is beneficial for complex multifactor experiments. edgeR generated an output of log2-fold change (log2FC) in expression, p values, and false discovery rates (FDRs) for every gene of each cluster.

#### Pathway analysis

Ingenuity Pathway Analysis (QIAGEN) was used to identify pathways that are up- or downregulated in each cluster between experimental conditions. Log2FC values, p values, and FDRs for each gene for each cluster were uploaded to the software. Results were confirmed with DAVID functional annotation (data not shown) (Huang et al., 2009, 2019).

#### Fluorescence *in situ* RNA hybridization for mouse tissue

Brains were postfixed in 4% PFA solution overnight then cryoprotected in 30% sucrose solution in PBS for up to 24h. Tissue was covered with optimal cutting temperature (OCT) media then sliced at 16 μm thickness using a Leica CM1950 cryostat directly onto Superfrost Plus slides (ThermoScientific) in an RNase free environment. Slides were then stored at −80°C. Sections were sliced in the coronal plane from Bregma −1.58 to −2.30 mm (Paxinos and Franklin, 2001).

Fluorescence multiplex *in situ* RNA hybridization (FISH) was performed as previously described using RNAscope technology (Bayraktar et al., 2018; Wang et al., 2012). After epitope retrieval and dehydration, sections on slides were processed for multiplexed FISH using the RNAScope LS Multiplex Assay (Advanced Cell Diagnostics) followed by immunohistochemistry on a Bond RX robotic stainer (Leica). Samples were first permeabilised with heat in Bond Epitope Retrieval solution 2 (pH 9.0, Leica - AR9640) at 95°C for 2 min, incubated in protease reagent (Advanced Cell Diagnostics) at 42°C for 10 min, and finally treated with hydrogen peroxide for 10 min to inactivate endogenous peroxidases and the protease reagent. Samples were then incubated in z-probe mixtures (*Pdgfra* 1:1, *Bmp4* 1:50, *Tcf7l2* 1:50 and *Plp1* 1:400) for 2 h at 42°C and washed 3 times. DNA amplification trees were built through incubations in AMP1 (preamplifier), AMP2 (background reducer), then AMP3 (amplifier) reagents (Leica) for 15-30 min each at 42°C. Between incubations, slides were washed with LS Rinse buffer (Leica). After, samples were incubated in channel-specific horseradish peroxidase (HRP) reagents for 15 min at 42°C, tyramide signal amplification (TSA) biotin or TSA fluorophores for 30 min and HRP blocking reagent for 15 min at 42°C. The following TSA labels were used to visualize z-probes: Atto 425-streptavidin (Sigma - 40709, 1:200), Opal 520 (1:500), Opal 570 (1:500), and Opal 650 (1:2500) fluorophores (Perkin Elmer).

Directly following the FISH assay, tissue was incubated with anti-Sox10 antibody in blocking solution for 1 hour (Abcam - AF2864, 1:100). To develop the antibody signal, samples were incubated in donkey anti-goat HRP (Thermo fisher Scientific - A15999, 1:200) for 1 hour, TSA biotin (Perkin Elmer - NEL700A001KT, 1:200) for 10 min and streptavidin-conjugated Alexa 700-streptavidin (Sigma - S21383, 1:200) for 30 min.

#### Human tissue

59h after death, hypothalamic samples were collected and stored in 10% neutral buffered formalin at room temperature for 24h, transferred to 70% ethanol, and processed into paraffin. 6 μm sections were cut and mounted onto Superfrost Plus slides (Thermo-Fisher Scientific) in an RNase free environment, and then dried overnight at 37°C.

Using a Bond RX robotic stainer (Leica), slides were deparaffinized, rehydrated, treated with Epitope Retrieval solution 2 88°C for 15 min, and with ACD Enzyme from the Multiplex Reagent kit at 40°C for 10 min. Z-probes (PDGFRA 1:50, ACD - 604488-C4; BCAS1, 1:50, ACD - 525788-C3; PLP1, 1:1, ACD - 499278) were used to detect mRNA transcripts in the tissue. Probe hybridization and signal amplification was performed according to manufacturer’s instructions. The following TSA labels were used to visualize z-probes: TSA plus-Cy5 (1:750 to detect PLP1, Akoya Biosciences - NEL745001KT), TSA plus-Fluorescein (1:300 to detect BCAS1, Akoya Biosciences - NEL741001KT) and Opal 620 (1:300 to detect PDGFRA, Akoya Biosciences - FP1495001KT). After completion of the FISH assay, slides were removed from the Bond RX and mounted using Prolong Diamond (ThermoFisher - P36965). We thank Julia Jones at the histopathology/ISH core facility at Cancer Research UK- Cambridge Institute for assistance with *in situ* hybridization.

#### Immunofluorescence

Brains were postfixed in 4% PFA solution overnight then cryoprotected in 30% sucrose solution in PBS for up to 24h. Tissue was covered with optimal cutting temperature (OCT) media (CellPath), and 30 μm thick coronal sections were obtained from Bregma 0.62 to −7.76 mm (Paxinos and Franklin, 2001) using a Bright Series 8000 sledge microtome.

Antigen retrieval was used for all experiments prior to antibody incubation. Sections were incubated in 10 mM sodium citrate (Fisher Scientific) in distilled water at 80°C for 20min then washed 3 times in PBS. For stains including the BrdU antibody, sections were then incubated in 2 N hydrochloric acid (Sigma) in distilled water at 37°C for 30min. The acid was neutralized by washing sections in 0.1 M sodium tetraborate (Sigma) in distilled water with hydrochloric acid to adjust pH (final pH = 8.5) for 10min, then sections were washed 3 times in PBS. For all experiments, sections were blocked in normal donkey serum (NDS, Vector Biolabs) in PBS plus 0.3% Triton X-100 (Sigma-Aldrich, 0.3% PBST) for 1h prior to primary antibody incubation (antibodies were diluted in 0.3% PBST with or without block).

Sections were incubated in primary antibody solution for the appropriate time at 4°C, then washed and incubated with appropriate secondary antibodies (specificity confirmed in the ME with secondary alone controls, not shown) diluted at 1:500 in 0.3% PBST for 90min at RT. Sections were then washed and mounted on slides (Clarity) with mounting media containing 4′,6-diamidino-2-phenylindole (DAPI, Life Technologies Corporation) and covered with thickness 1.0 coverslips (Marienfeld).

#### Tissue clearing

Brains were postfixed in 4% PFA solution overnight. Whole brains were washed in PBS 2 times for 2h. Brains were trimmed in the coronal plane using a Leica VT1000s vibrotome until approximately Bregma −1.58 mm. Four to six 200 μm sections were then sliced from each brain and placed in a 24-well plate (Costar) in PBS. Tissue clearing was performed using the Clear, Unobstructed Brain/Body Imaging Cocktails (CUBIC) method as published (Susaki et al., 2014, 2015), with minor modifications.

#### Preparing reagents

The CUBIC1 solution containing 25% urea (VWR Chemicals – 443874G), 28.8% distilled water, 31.2% Quadrol (Aldrich - 122262, diluted to 80% in distilled water) and 15% Triton X-100 (Fisher Bioreagents – BP151) was made by mixing the first 3 components on a hot plate at 150°C for 15min. After cooling, Triton X-100 was added to the solution and mixed at RT. CUBIC2 solution is comprised of 25% urea, 50% saccharose (VWR Chemicals – 443815S), 15% distilled water, and 10% triethanolamine (Sigma – 90279). Urea, saccharose, and water were mixed on a hot plate at 150°C for 30min then allowed to cool to RT. Triethanolamine was then added to the solution and mixed at RT.

#### Tissue clearing – CUBIC1

PBS in the well plate was replaced with a mixture of 1:1 CUBIC1 solution and distilled water plus Hoechst stain (Life Technologies – H3570, 1:2000, 1ml per well). The seam of the well plate was sealed with Parafilm and the well plate was placed in a shaking waterbath at 37°C for 3h. The CUBIC1/water solution was then discarded and replaced with 100% CUBIC1 solution plus Hoechst and kept at 37°C overnight. The following day, the samples were washed in PBS three times for 1h on a shaker. The samples were then placed in 30% sucrose in PBS until the sections sank to the bottom of the wells. After, the sections were immersed in OCT and frozen at −80°C at least overnight.

#### IHC labeling of cleared tissue

The samples were thawed and washed in PBS three times for 1h on a shaker. The samples were placed in a new 24-well plate and covered with primary antibodies diluted in PBS plus 2% Triton X-100 (2% PBST) and 10% NDS for 48h at 4°C on a shaker. Samples were then washed in 0.3% PBST at RT three times for 1h. The samples were then placed in secondary antibodies diluted in 2% PBST and 10% NDS for 48h at 4°C on a shaker. Samples were then washed in 0.3% PBST at RT three times for 1h.

#### Matching refractive index – CUBIC2

IHC-labeled samples were immersed in a mixture of 1:1 CUBIC2 solution and PBS. The well plate was sealed with Parafilm then placed in a shaking waterbath at 37°C for 3h. The CUBIC2/water solution was then discarded and replaced with 100% CUBIC2 solution and kept at 37°C at least overnight, but maximum 72h. The day before imaging, samples were placed in a 1:1 mixture of mineral oil (Sigma - M8410) and silicone oil (Sigma - 175633).

#### Resin embedding

Brains were postfixed in 4% glutaraldehyde for 24h then moved to PBS. 1 mm-thick sections containing the ME were sliced by hand from the brains and were stained with 2% osmium tetroxide (Oxkem) for 24h at 4°C. Sections were washed with water 3 times then dehydrated using an ethanol gradient as follows, on a rotator: 50% 2 times for 15min, 70% 2 times for 15min, 90% 2 times for 15min, 95% 2 times for 15min, 100% 3 times for 10min. Sections were then placed in propylene oxide (Agar Scientific) for 20 min. Sections were incubated in a mixture of 1:1 propylene oxide and resin (TAAB) for 6h on a rotator, then in 100% resin for 24h. Sections were mounted in resin in plastic molds (TAAB) and incubated at 60°C for 24h.

#### Toluidine blue labeling

Resin blocks were trimmed with a microtome (Leica RM-2065) to expose the tissue then 0.75 μm-thick sections were placed on a water droplet on a slide. Slides were heated on a hotplate to evaporate the water, then toluidine blue (0.5%, Merck) was applied to the sections for 30 s before washing off with distilled water.

#### Post staining for transmission electron microscopy

Resin embedded tissues were trimmed around the ME and semi thick slices were cut to create sections that only contained the ME. Then, 70nm ultrathin sections were sliced on an ultramicrotome (Reichert-Jung - Ultra-cut 701701 Ultra Microtome) with a diamond knife (Diatome - Ultra 45). Sections were placed on mesh copper grids (size 300) and were post stained with aqueous uranyl acetate for 6 min then lead citrate for 2 min.

#### Confocal microscopy

Thin immunolabelled mouse sections (63x and 40x oil objective) and human tissue labeled with FISH (10x dry and 20x oil objectives) were imaged using a Leica SP8 confocal microscope. For mouse tissue, sections were imaged at multiple points in the z plane (z stacks) at intervals of 3.3 μm to collect signal from the entire depth of the tissue for the region of interest (ROI). ROIs for mouse tissue included the vascular organ of the lamina terminalis, subfornical organ, CC and area postrema. For human tissue, sections were imaged at multiple points in the z plane (z stacks) at intervals of 5 μm or 0.5 μm for volumetric analysis. ROIs for human tissue were the ME and ARH at 20x and the full coronal hypothalamic section at 10x. Gain and laser power settings remained the same between experimental and control conditions within each experiment.

#### High-content confocal microscopy

Mouse tissue labeled with FISH was imaged using a spinning disk Operetta CLS (Perkin Elmer). Sections were imaged in confocal mode using a sCMOS camera and a 40x automated-water dispensing objective. Sections were imaged with z stacks at intervals of 1 μm. ROIs included the ME and CC. Gain and laser power settings remained the same between experimental and control conditions within each experiment.

#### Light microscopy

Toluidine blue-labeled tissue was imaged using a (Nikon Eclipse E600) light microscope with 40x and 100x (dry) objectives. The ROI was the ME.

#### Spinning disk confocal microscopy

Thick cleared mouse tissue was imaged using an Andor Dragonfly spinning disk confocal with a 20x objective. Sections were placed in a glass-bottom dish (MatTek – P-35G-0-14-C) with a small amount of 1:1 oil mixture to coat the interface of the glass and tissue. Sections were imaged with z stacks at the software recommended interval. The ROI was the ME and ARH – the large field of view of this microscope allowed both structures to be imaged at once without tiling.

#### Image analysis

For all experiments, the images were blinded to experimental condition before quantification.

#### Mouse FISH

Harmony software (Perkin Elmer) was used to automatically quantify number of labeled RNA molecules (spots) per cell, intensities of spots, and area of spots.

#### Immunohistochemistry, human FISH, toluidine blue labeling

For immunolabelled, toluidine blue labeled mouse sections and FISH labeled human sections, Fiji software was used to analyze colocalization, distribution, and counts/density of markers (Schindelin et al., 2012). For images in z stacks, individual images were first projected into a single image (a ‘Z-Project’ at maximum intensity) so all cells could be counted at once, and to eliminate double-counting. Areas of ROIs were measured by setting the image scale according to scale bars imprinted on images during acquisition, then tracing the ROI with the freehand tool and measuring. Borders of the ME, ARH, and other ROIs were determined using the Paxinos and Franklin Mouse Brain Atlas (Paxinos and Franklin, 2001). The Fiji manual cell counter was used to count marker-positive cells or axons. To quantify cell size, 10 cells per section were measured by tracing their perimeters with the Fiji freehand tool.

#### Synaptic puncta quantification

Stacks of microphotographs from confocal microscopy were analyzed with Imaris software (Bitplane). Surface rendering was done for each channel prior to contact analysis. Contacts between synapses and OPC somas and elongations were determined using the “Surface Surface Contact Area” plug-in (Imaris, Bitplane). Numbers of contacts between VGAT-positive elements and VGluT1-positive elements were counted on each PDGFR α-positive elements of OPC cells. Scores were standardized by calculating the ratio between VGluT1-positive elements and VGAT-positive elements per OPC cell.

#### TNR and WFA staining quantification

20um stacks taken at 1um intervals were analyzed using Imaris software (Bitplane). Surfaces were reconstructed in 3D for each staining. Surface, volume and mean intensity were exported for each TNR+ and WFA+ object.

#### Cleared tissue visualization

Videos of thick cleared tissue were made using Imaris software (Oxford Instruments).

#### Myelin thickness

To assess differences in g-ratio between fasted and refed conditions, at least 100 distinct transverse axons were measured per animal. Using Fiji, the cross-sectional area of axons was measured by tracing the outside of the axon with the freehand tool. Similarly, the outside of the myelin sheath was traced to determine the area of the myelin+axon. Diameters of the axon and the myelin+axon were back-calculated from the areas, assuming the cross sections were perfect circles. The g-ratio was then calculated by dividing the axon diameter by the myelin+axon diameter.

### Quantification and statistical analysis

All code used for analyzing data and creating figures related to scRNaseq is found in the online repository Github: Kohnke-et-al-2019. Ranking of cluster-defining genes and statistical significance of differentially expressed genes (DEGs) was determined by the cellrangerRkit (10x) and edgeR (McCarthy et al., 2012; Robinson et al., 2010) packages in R. Statistical significance of pathways changed and upstream regulators of DEGs between fasting and refeeding was determined using Ingenuity Pathway Analysis (QIAGEN). Figures relating to scRNaseq data were created using the cellrangerRkit, ggplot2, tidyr, and GOplot packages (Walter et al., 2015; Wickham, 2014, 2016; Wilcox, 2005). For histology experiments, GraphPad Prism 8 (GraphPad Software) was used to define statistical significance (p ≤ 0.05) and create graphs. Two-sided unpaired Student’s t tests were used to compare data between fasted and refed conditions (averages per animal) and ANCOVA was used to compare slopes of linear regression lines. All data are expressed as mean ± SEM. Statistical details of experiments and p values can be found on figures and figure legends.

## Supplemental information

Document S1. Figures S1–S6

Table S1. Top 50 defining genes per cluster of ME cell types, related to Figures 1 and 3

Table S2. Top 50 differentially expressed genes in ME cell types (fasted versus refed), related to Figures 1 and 3

Table S3. Pathways significantly changed between fasting and refeeding in OL lineage cells, related to Figure 3

Table S4. Refeeding regulates pathways associated with OL proliferation, differentiation, cell cycle progression, and myelination, related to Figure 3

Table S5. Upstream regulators of differentially expressed genes, related to Figure 3

Document S2. Article plus supplemental information
