# Supplementary material for: Nutritional regulation of oligodendrocyte differentiation regulates perineuronal net remodeling in the median eminence
Source: Cell Rep. 2021 Jul 13;36(2):109362. doi: 10.1016/j.celrep.2021.109362 (PMC8293628; doi:10.1016/j.celrep.2021.109362)
Supplement: Document S1. Figures S1–S6 [file mmc1.pdf]

Cell Reports, Volume *36*

Supplemental information

Nutritional regulation of oligodendrocyte differentiation regulates perineuronal net remodeling in the median eminence

Sara Kohnke, Sophie Buller, Danae Nuzzaci, Katherine Ridley, Brian Lam, Helena Pivonkova, Marie A. Bentsen, Kimberly M. Alonge, Chao Zhao, John Tadross, Staffan Holmqvist, Takahiro Shimizo, Hannah Hathaway, Huiliang Li, Wendy Macklin, Michael W. Schwartz, William D. Richardson, Giles S.H. Yeo, Robin J.M. Franklin, Ragnhildur T. Karadottir, David H. Rowitch, and Clemence Blouet

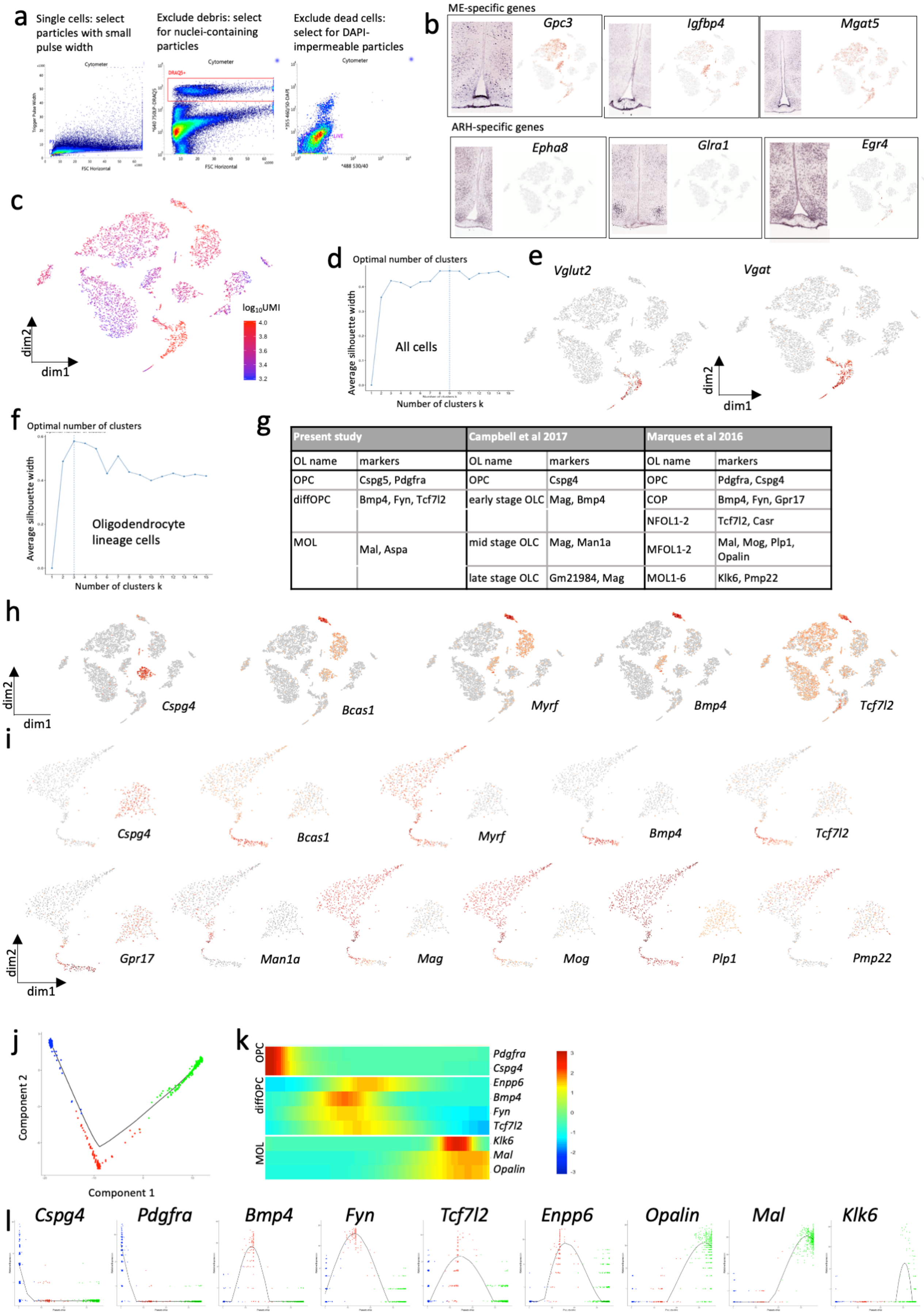

Present study		Campbell et al 2017		Marques et al 2016	
OL name	markers	OL name	markers	OL name	markers
OPC	Cspg5, Pdgfra	OPC	Cspg4	OPC	Pdgfra, Cspg4
diffOPC	Bmp4, Fyn, Tcf7l2	early stage OLC	Mag, Bmp4	COP	Bmp4, Fyn, Gpr17
				NFOL1-2	Tcf7l2, Casr
MOL	Mal, Aspa	mid stage OLC	Mag, Man1a	MFOL1-2	Mal, Mog, Plp1, Opalin
		late stage OLC	Gm21984, Mag	MOL1-6	Klk6, Pmp22

Figure S1. Single cell transcriptomic analysis reveals 3 types of oligodendrocyte lineage cells in the ME. Related to Figure 1.

(a) FACS cell sorting strategy. **(b)** Allen Mouse Brain ISH gene expression images compared to gene expression in current dataset for ME / ARC specific genes (red labels high expression of the gene in the tSNE plot). **(c)** Log_{10}UMI counts per cell mapped on tSNE plot. **(d)** Silhouette analysis (optimal cluster analysis) of tSNE object coordinates. **(e)** GABAergic (express vesicular GABA transporter, Vgat) and glutamatergic (express vesicular glutamate transporter 2, Vglut2) gene expression in current dataset. **(f)** Silhouette analysis (optimal cluster analysis) of tSNE object coordinates. **(g)** Comparison of terminology and defining markers between current study and two others examining single-cell transcriptomes of OLs. **(h-i)** tSNE plots of current dataset (main and oligodendrocyte lineage specific clustering) showing expression of defining OL markers used in other studies. Red = high expression, grey = low expression. **(j)** Unsupervised ordering of OL clusters along a developmental trajectory (arrows indicate direction) show developmental progression from OPCs (blue) to NFOLs (red) to MOLs (green). **(k)** Heatmap showing relative expression of genes associated with OPCs, NFOLs, and MOLs along pseudotime. **(l)** Scatterplots showing relative expression of genes associated with OPCs, NFOLs, and MOLs along pseudotime.

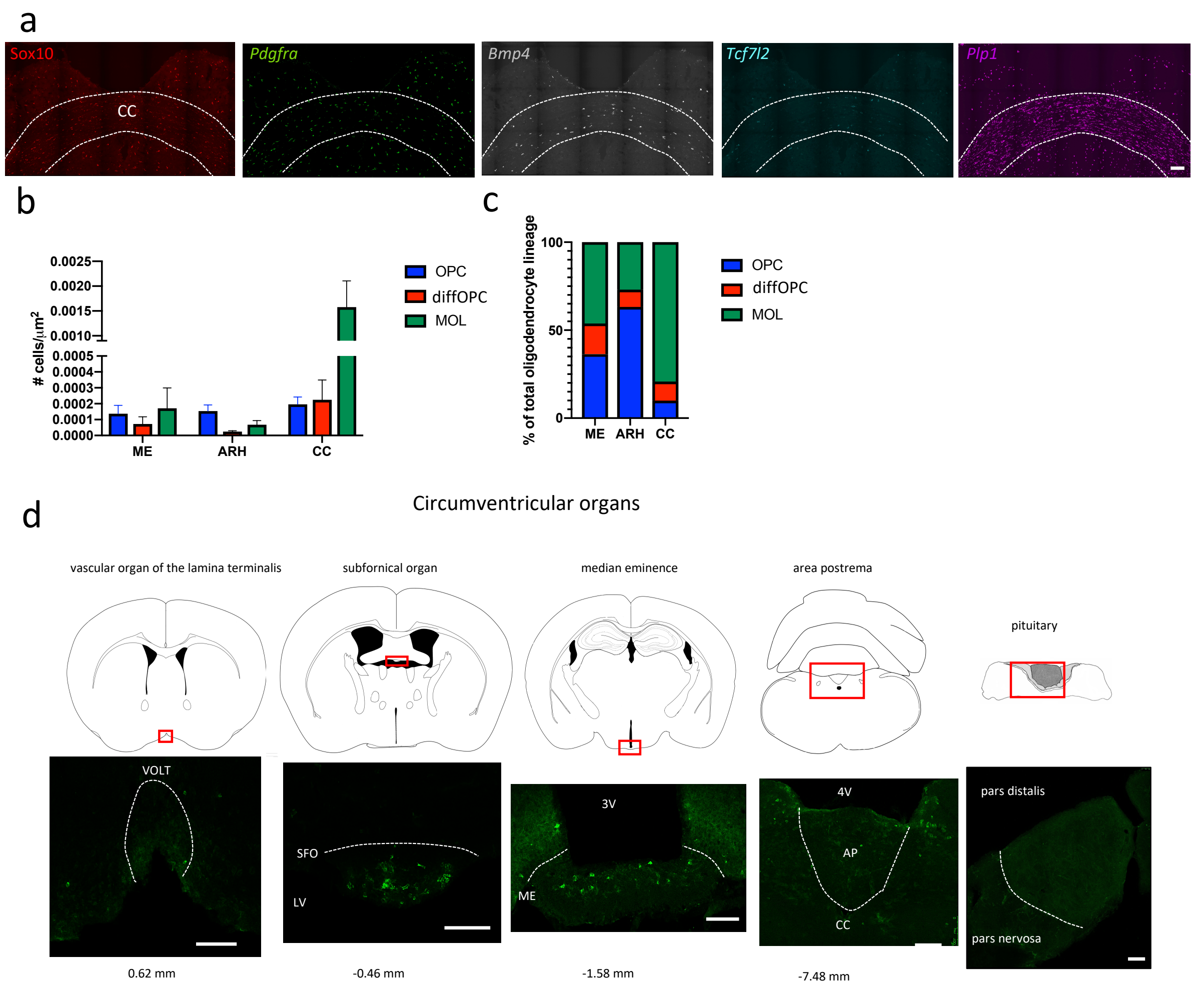

Figure

Figure S2. The diffOPC and MOL populations are concentrated in the dorsal part of the murine and human ME. Related to Figure 2.

(a) RNAscope combined to immunohistochemistry enables detection of OL subtype marker gene expression in the corpus callosum: red = Sox10 (IHC, pan-OL marker), green = *Pdgfra* (OPC marker), grey = *Bmp4* (NFOL marker), blue = *Tcf7l2* (NFOL marker), purple = *Plp1* (MOL marker). Scale bar = 100 µm. (b) Densities of OL lineage cells in the ME, ARH and CC. Error bars depict mean ± SEM. (c) Proportions of OPCs/NFOLs/MOLs in the ME, ARC, and CC. (d) APC (green, a postmitotic OL marker) labels sparse cells in the SFO and AP but not in the VOLT or pituitary. Scale bars = 100 µm

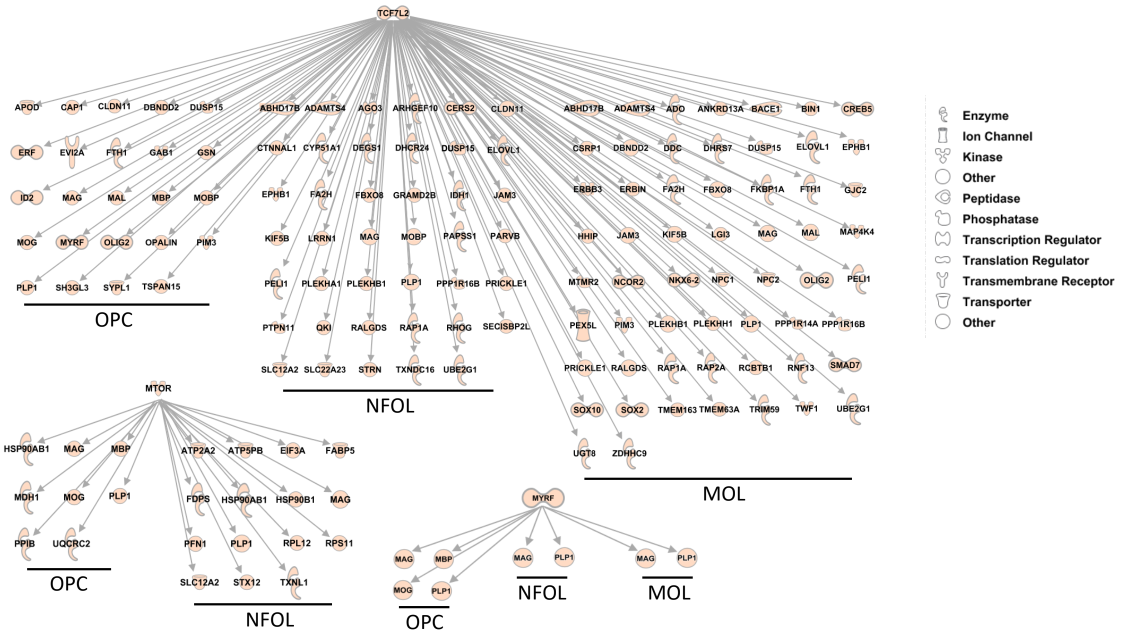

Figure S3. Nutritional signals rapidly regulate the transcriptome of oligodendrocyte lineage cells in the adult ME. Related to Figure 3.

Genes downstream from top upstream regulators *Tcf7l2, Mtor*, and *Myrf*

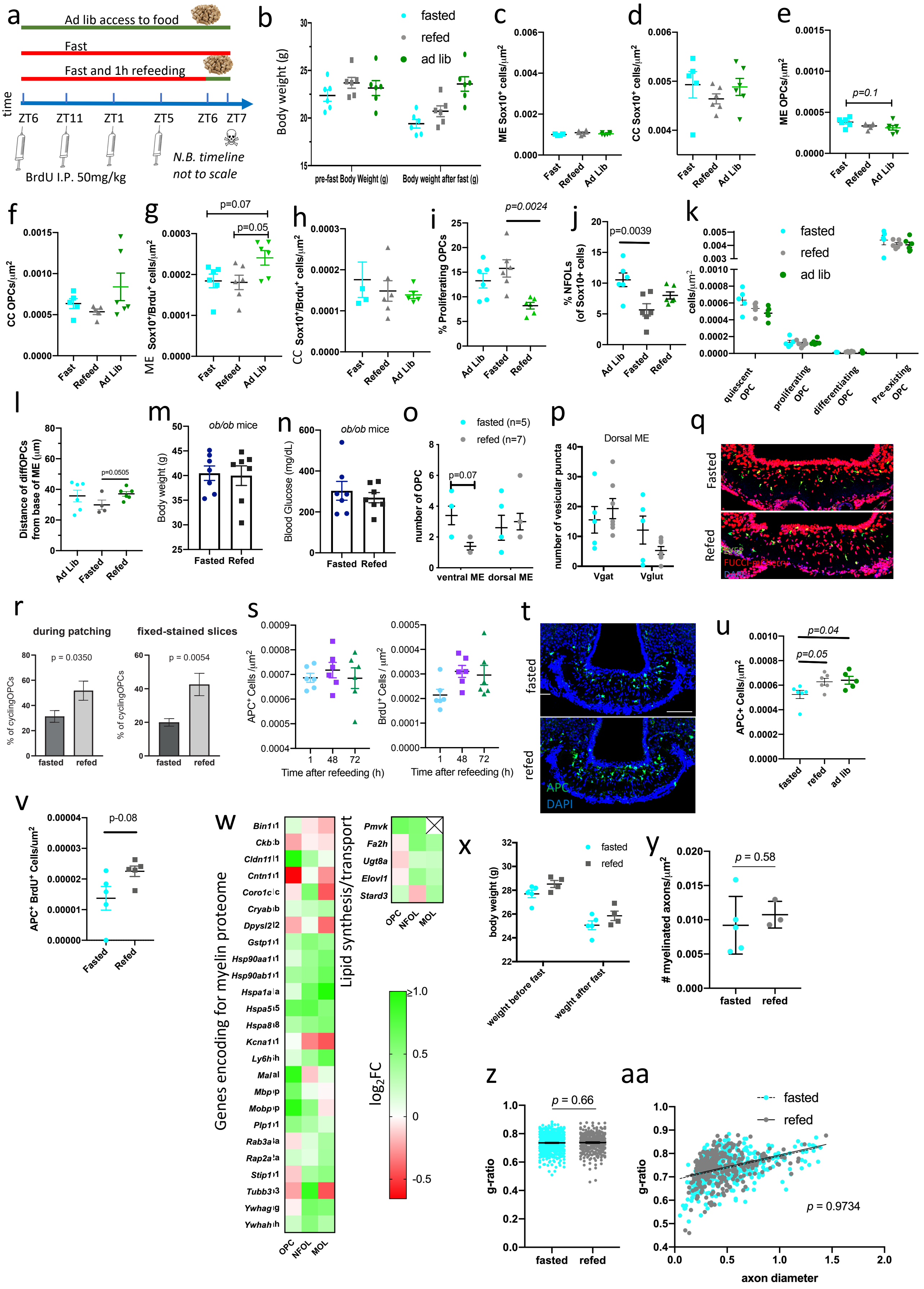
a
Ad lib access to food
Fast
Fast and 1h refeeding
time
ZT6 ZT11 ZT1 ZT5 ZT6 ZT7
BrdU I.P. 50mg/kg
N.B. timeline not to scale
b
fasted
refed
ad lib
Body weight (g)
pre-fast Body Weight (g)
Body weight after fast (g)
c
ME Sox10+ cells/μm2
Fast Refeed Ad Lib
d
CC Sox10+ cells/μm2
e
ME OPCs/μm2
p=0.1
f
CC OPCs/μm2
g
p=0.07
p=0.05
ME Sox10+/Brdu+ cells/μm2
h
CC Sox10+/Brdu+ cells/μm2
i
p=0.0024
% Proliferating OPCs
Ad Lib Fasted Refed
j
p=0.0039
% NFOLs (of Sox10+ cells)
k
fasted
refed
ad lib
cells/μm2
quiescent OPC
proliferating OPC
differentiating OPC
Pre-existing OPC
l
Distance of diffOPCs from base of ME (μm)
p=0.0505
m
ob/ob mice
Body weight (g)
Fasted Refed
n
ob/ob mice
Blood Glucose (mg/dL)
o
fasted (n=5)
refed (n=7)
number of OPC
p=0.07
ventral ME dorsal ME
p
Dorsal ME
number of vesicular puncta
Vgat Vglut
q
Fasted
Refed
YFP
FUCCI-mKO
DAPI
r
during patching
fixed-stained slices
p = 0.0350
p = 0.0054
% of cyclingOPCs
fasted refed
s
APC+ Cells /μm2
BrdU+ Cells / μm2
Time after refeeding (h)
1 48 72
t
fasted
refed
APC
DAPI
u
p=0.04
p=0.05
APC+ Cells/μm2
fasted refed ad lib
v
p-0.08
APC+ BrdU+ Cells/μm2
Fasted Refed
w
Genes encoding for myelin proteome
Bin1 Ckb Cldn11 Cntn1 Coro1c Cryab Dpysl2 Gstp1 Hsp90aa1 Hsp90ab1 Hspa1a Hspa5 Hspa8 Kcna1 Ly6h Mal Mbp Mobp Plp1 Rab3a Rap2a Stip1 Tubb3 Ywhag Ywhah
Lipid synthesis/transport
Pmvk Fa2h Ugt8a Elovl1 Stard3
OPC NFOL MOL
log2FC
≥1.0
0.5
0
-0.5
x
fasted
refed
body weight (g)
weight before fast
weght after fast
y
p = 0.58
# myelinated axons/μm2
fasted refed
z
p = 0.66
g-ratio
fasted refed
aa
fasted
refed
g-ratio
p = 0.9734
axon diameter

Figure S4. Nutritional signals rapidly regulate OPC proliferation and differentiation in the ME. Related to Figure 4.

(a-l) Brdu incorporation into OL lineage cells during fasting-refeeding**. (a)** Brdu administration paradigm. **(b)** Body weights before and after the 24h fast. Density of $Sox10^+$ cells **(c,d)**, OPCs **(e,f)**, $Sox10^+/Brdu^+$ cells **(g,h)** in the ME **(c,e,g)** and CC **(d,f,h)**. Percentages of OPCs **(i)**, diffOPCs **(j)** in the ME. **(k)** Density of cells at different stages of the OL lineage in the CC. **(l)** Dorsoventral localization of diffOPCs ($Brdu^+$, $Sox10^+$, $Pdgfra^-$ cells) in the ME. **(m)** Body weight and **(n)** blood glucose of *ob/ob* mice. **(o)** Distribution of OPCs in the ventral and dorsal ME. **(p)** Quantification of GABAergic and glutamatergic vesicular puncta in the ventral ME. **(q)** YFP (green), FUCCI-mcherry (red), dapi (blue) immunostaining in the ME of fasted and refed *NG2-EYFP:FUCCI2a* mice. Scale bar: 100 um **(r)** Quantification of quiescent and cycling OPCs in fasted and refed *NG2-EYFP:FUCCI2a* mice. **(s)** Density of APC^+ MOLs and $Brdu^+$ cells 1h, 48h and 72h after refeeding. **(t)** Immunodetection of APC in the ME of fasted and refed mice and **(u)** quantification of APC^+ OLs in the ME of fed, fasted and refed mice. Scale bar: 100 um **(v)** Density of $Brdu^+$ MOLs (APC+) in the ME of fasted and refed Brdu treated mice. **(w)** Log_2FC genes significantly changed between the fasted and refed conditions ($p < 0.05$ and FDR < 0.25 for at least one cluster) and part of the myelin proteome or involved in lipid synthesis and transport. An 'X' indicates the gene is not expressed in one or both conditions in that OL cluster. **(x)** Body weights before and after the overnight 16h fast. **(y)** Measurements of density of myelinated axons of animals fasted (n = 5) and those fasted then refed 2h (n = 3), **(z)** Measurements of g-ratio of myelinated axons in fasted (n = 5 mice, 481 axons) and refed (n = 3 mice, 308 axons) conditions. **(aa)** Measurement of slopes of linear regression lines to fit g-ratio (as in f) plotted against axon diameter. Error bars indicate mean ± SEM

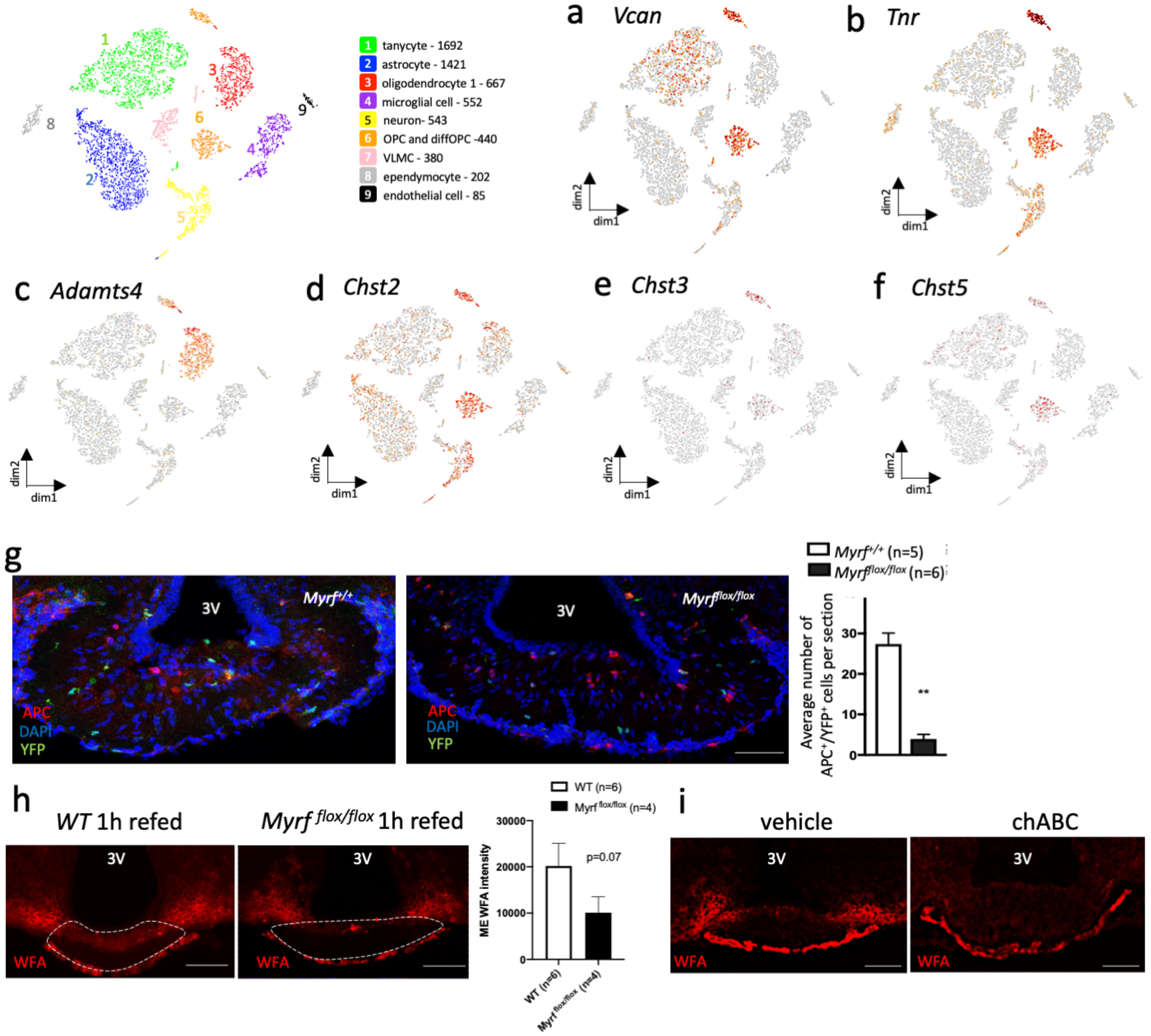

Figure S5. Nutritional regulation of oligodendrocyte lineage progression in the ME regulates local perineuronal nets. Related to Figure 5.

(a-f) Heatmap of genes involved in ECM assembly and remodelling and enriched in OL lineage cells**. (g)** Co-staining of APC (red), YFP (green) and DAPI (blue) in the ME of *Myrf*$^{+/+}$ and *Myrf*$^{flox/flox}$ mice, and quantification of the average number of APC^{+}/YFP^{+} cells per section (n=4). **: p<0.01. scale bar : 75 µm. **(h)** WFA immunolabelling in 1h refed WT or *Myrf*$^{flox/flox}$ mice. Circled areas depicts ME are quantified for WFA intensity. Scale bar : 100 µm. (n=4) **(i)** WFA immunolabelling in the median eminence of mice treated locally with vehicle (saline) or chABC. Scale bar : 100 µm. Data are means +sem.

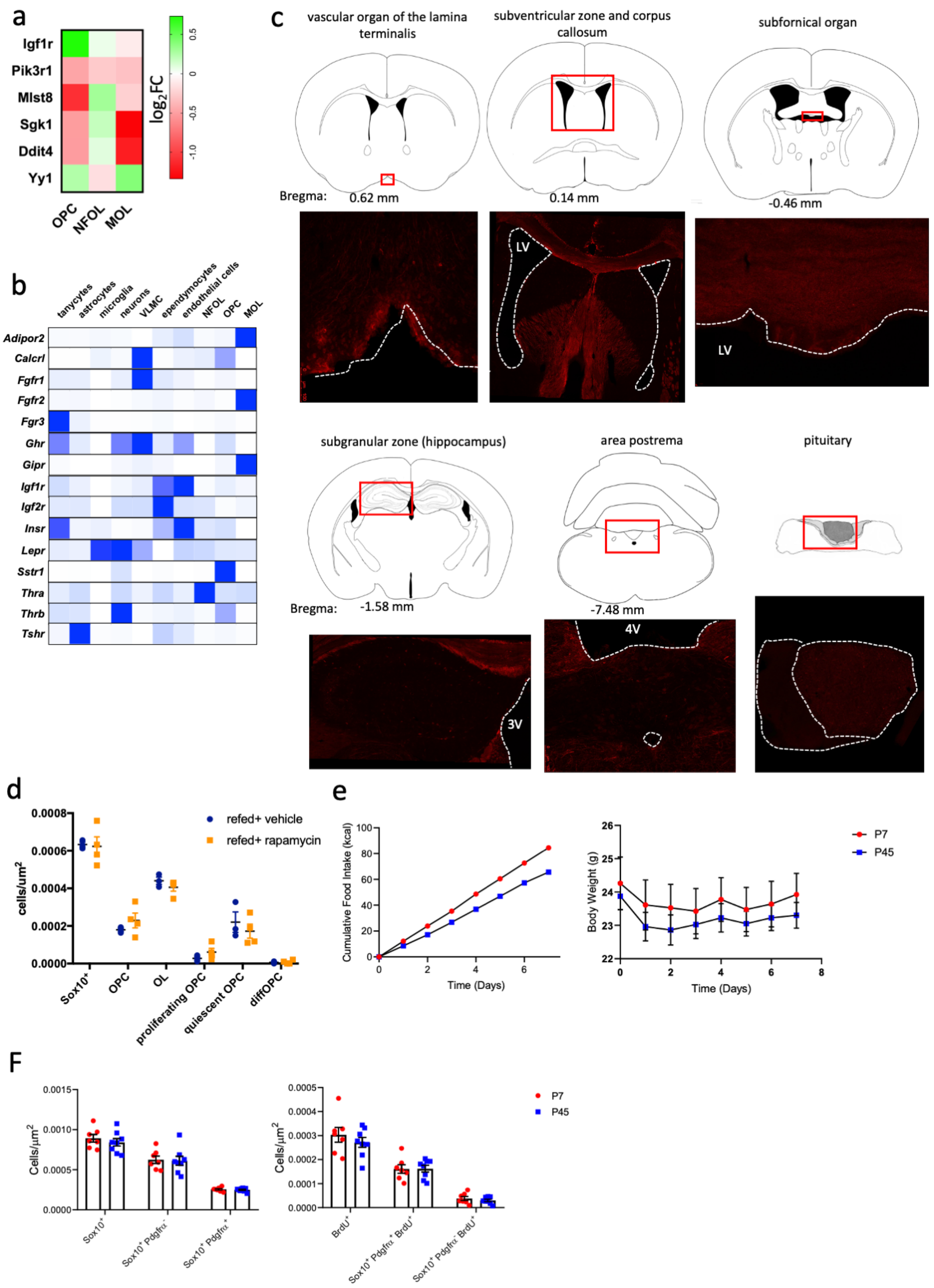

Figure S6. mTORC1 signaling is highly active and nutritionally regulated in the ME oligodendrocytes. Related to Figure 6.

(a) Heatmap of genes involved in mTOR signaling that are differentially expressed in scRNAseq dataset ($p < 0.5$, FDR < 0.25, *Pik3r* produces the p85 protein). **(b)** Relative expression of receptors for metabolic hormones in various cell types present in the ME. Scale is variable. **(c)** Labelling with the antibody to pmTOR (Ser 2448) in white matter tracts and other circumventricular organs throughout the mouse brain. **(d)** ME density of cells in the OL lineage in 1h refed mice pretearted with vehicle or rapamycin to inhibit mTORC1. **(e)** Cumulative food intake and body weight gain in mice fed isocaloric low-protein (P7) or high protein (P45) diets for 7 days. (n=8) **(f)** ME density of cells in the OL lineage and cells that incorporated BrdU during 24h in mice fed with isocaloric low-protein (P7) or high protein (P45) diets. Data are means+sem.
